# Establishment of induced pluripotent stem cells from normal B cells and inducing AID expression in their differentiation into hematopoietic progenitor cells

**DOI:** 10.1038/s41598-017-01627-1

**Published:** 2017-05-10

**Authors:** Fumihiko Kawamura, Makoto Inaki, Atsushi Katafuchi, Yu Abe, Naohiro Tsuyama, Yumiko Kurosu, Aki Yanagi, Mitsunori Higuchi, Satoshi Muto, Takumi Yamaura, Hiroyuki Suzuki, Hideyoshi Noji, Shinichi Suzuki, Mitsuaki A. Yoshida, Megumi Sasatani, Kenji Kamiya, Masafumi Onodera, Akira Sakai

**Affiliations:** 1 Department of Radiation Life Sciences, Fukushima Medical University School of Medicine, Fukushima, Japan; 2 Department of Genetics, National Research Institute for Child Health and Development, Tokyo, Japan; 3 Department of Regenerative Surgery, Fukushima Medical University School of Medicine, Fukushima, Japan; 4 Department of Medical Oncology, Fukushima Medical University School of Medicine, Fukushima, Japan; 5 Department of Thyroid and Endocrinology, Fukushima Medical University School of Medicine, Fukushima, Japan; 6 Department of Radiation Biology, Institute of Radiation Emergency Medicine, Hirosaki University, Hirosaki, Japan; 7 Department of Experimental Oncology, Research Institute for Radiation Biology and Medicine, Hiroshima University, Hiroshima, Japan

## Abstract

B cell derived induced pluripotent stem cells (BiPSCs) were recently established from peripheral blood B cells by the simultaneous transfection of Yamanaka factors (Oct3/4, Sox2, Klf4, c-Myc) and C/EBPα using a Sendai virus vector. Here, using a different method, we established BiPSCs with immunoglobulin heavy chain (IgH) gene rearrangement from normal B cells purified from lymph nodes. The critical points of our method are pre-stimulation of B cells with IL-21 and CD40-ligand (CD40L), followed by consecutive transfection of highly concentrated Yamanaka factors using a retroviral vector. Following each transfection the cells were centrifuged onto a retronectin coated plate and the activated by IL-4, IL-2, and CD40L. Furthermore, we established BiPSCs (BiPSC-A) in which activation-induced cytidine deaminase (AID) could be induced using the doxycycline-controlled. Both the parental BiPSC and BiPSC-A showed the capability of differentiating into hematopoietic progenitor cells (HPCs) based on confirmation of CD34 expression and colony-formation from CD34-positive cells. The findings that BiPSC-A can differentiate into HPCs suggest that there is a possibility that induction of AID expression would result in chromosomal translocations in the process of differentiation from BiPSCs, and therefore that these BiPSCs could be useful in elucidating the tumor origin of abnormal B cells in myelomagenesis.

## Introduction

Somatic cells can be reprogrammed into induced pluripotent stem cells (iPSCs)^1^ by exogenous expression of reprogramming factors (Yamanaka factors) such as Oct4, Sox2, Klf4 and c-Myc. Since the invention of this method, iPSCs have been established from a variety of somatic cells not only for regenerative medicine but also for studies of the pathogenesis of inherited genetic disease^2–5^ or neoplasms^6–9^. In term of the establishment of iPSCs from blood cells, the T cells that were derived from antigen-specific CD8^+^ T cells in an HIV-1-infected patient^10^, or from mature cytotoxic T cells that were specific for the melanoma epitope MART-1^11^, were reprogramed into iPSC, and were then re-differentiated into CD8^+^ cells that possessed antigen-specific killing activity for treatment of patients with AIDS or melanoma, respectively. The important point of these studies is that the rearrangement of the T cell receptor (TCR) of the established T cell derived iPSC (TiPSC) was the same as that of the original T cell. Similarly, if B cell derived iPSC (BiPSC) could be established from mature B cells or plasma cells and then be subsequently redifferentiated into mature B cells or plasma cells, it should be possible to make mature B cells that are specific for an antigen or make plasma cells that are producing monoclonal antibodies.

In a mouse system, chimeric mice were produced from iPSC that were established from mouse embryonic fibroblasts (MEFs). Subsequently, BiPSCs that had a B cell receptor (BCR) that was identical to that of B cells isolated from the chimeric mice were established by reactivation of Yamanaka factors together with either ectopic expression of the myeloid transcription factor CCAAT/enhancer-binding-protein-α (C/EBPα) or specific knockdown of the B cell transcription factor Pax5^12^. On the other hand, Wada *et al*. established iPSCs from MEFs and mouse splenic B cells, and both iPSCs differentiated into a T-cell lineage. However, these cells were relatively resistant to B-cell lineage differentiation and it was speculated that this resistance was due to a defect in Pax5 expression in the differentiated cells^13^. Recently, Stefano *et al*. reported that the overexpression of Tet2 induced by the transfection of C/EBPα enhances Yamanaka factor-induced B-cell reprograming^14^. Subsequently, BiPSCs were established from peripheral blood B cells by the simultaneous transfection of Yamanaka factors and C/EBPα using a Sendai virus vector^15^.

In the present study we were able to establish BiPSCs from normal B cells purified from lymph nodes by using a different method. Here, normal B cells were pre-activated with IL-21 and CD40-ligand (CD40L) and were then consecutively transfected on a retronectin coated plate with highly concentrated Yamanaka factors using a retroviral vector and centrifugation.

Activation-induced cytidine deaminase (AID) is an enzyme that initiates somatic hypermutation (SHM) and class-switch recombination (CSR)^16^. Not only immunoglobulin heavy chain (IgH) gene, but also gene mutations in B cells induced by AID, are supported as a cause of lymphomagenesis^17–22^. Recently, model mice with diffuse large B-cell lymphoma (DLBCL) were reported, in which DNA repair abnormalities after mutations or cleavages of DNA induced by AID-generated uracil-guanine (U-G) mismatch regulate malignant transformation, was reported^23^. Since BiPSCs established from this mouse have IgH gene rearrangement, we subsequently established BiPSCs in which AID expression could be induced by using a doxycycline-controlled system (tet-off system), in order to determine whether those BiPSCs would produce B cells with chromosomal translocation, which is thought to be one origin of B cell malignancies.

## Results

### Generation of iPS cells from CD19^+^ cells of human lymph nodes

**Figure 1:**
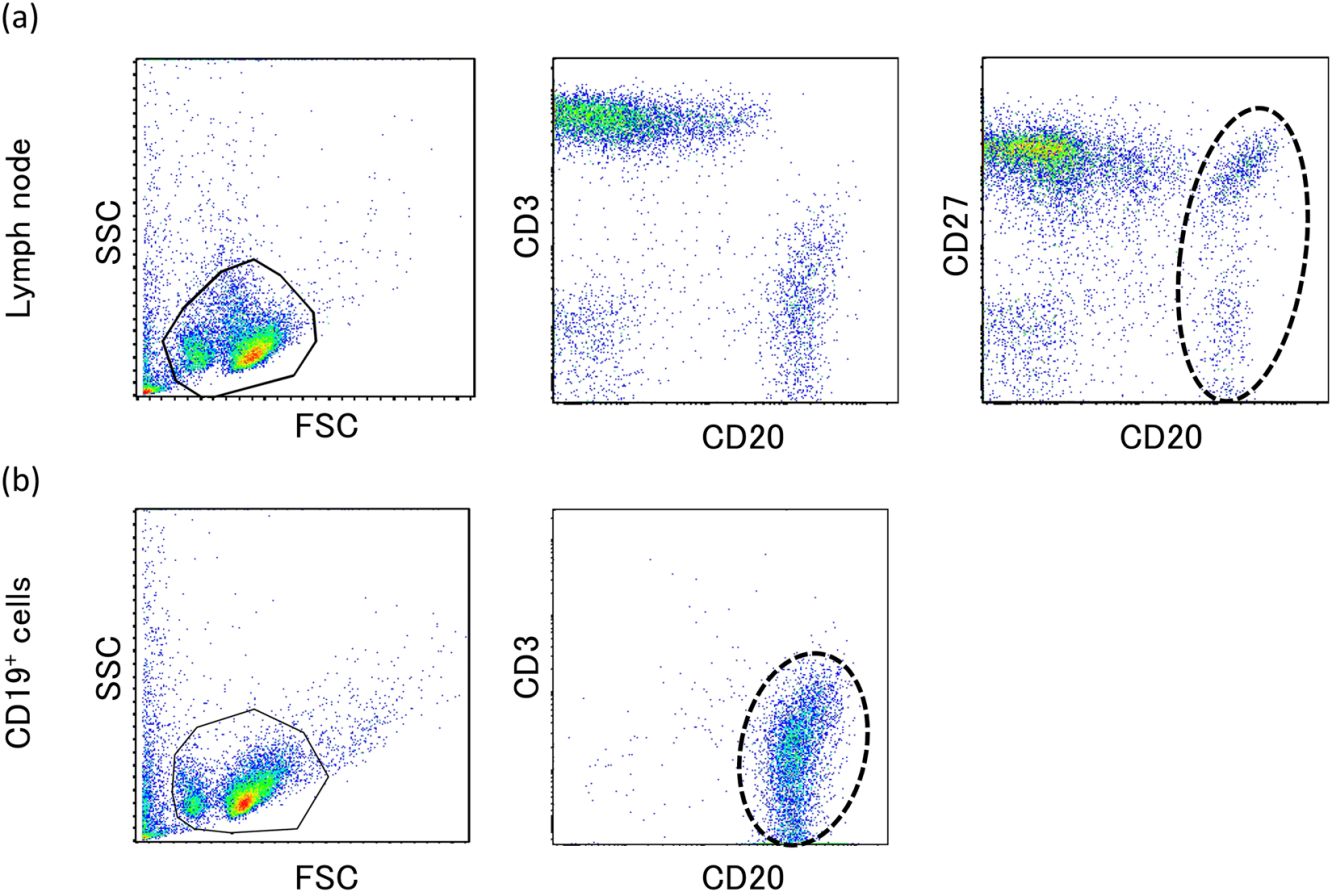
Cell surface antigen analysis by two-color flow cytometry. (**a**) Phenotype analysis of lymphocytes of the lymph node by flow cytometric analysis with a combination of anti-CD3/-CD20 and anti-CD27/-CD20 antibodies. B cells are surrounded by a dotted line. (**b**) Phenotype analysis of B cells purified using CD19-microbeads by flow cytomeric analysis with a combination of anti-CD3/-CD20 antibodies. Purified B cells are surrounded by a dotted line.

**Figure 2:**
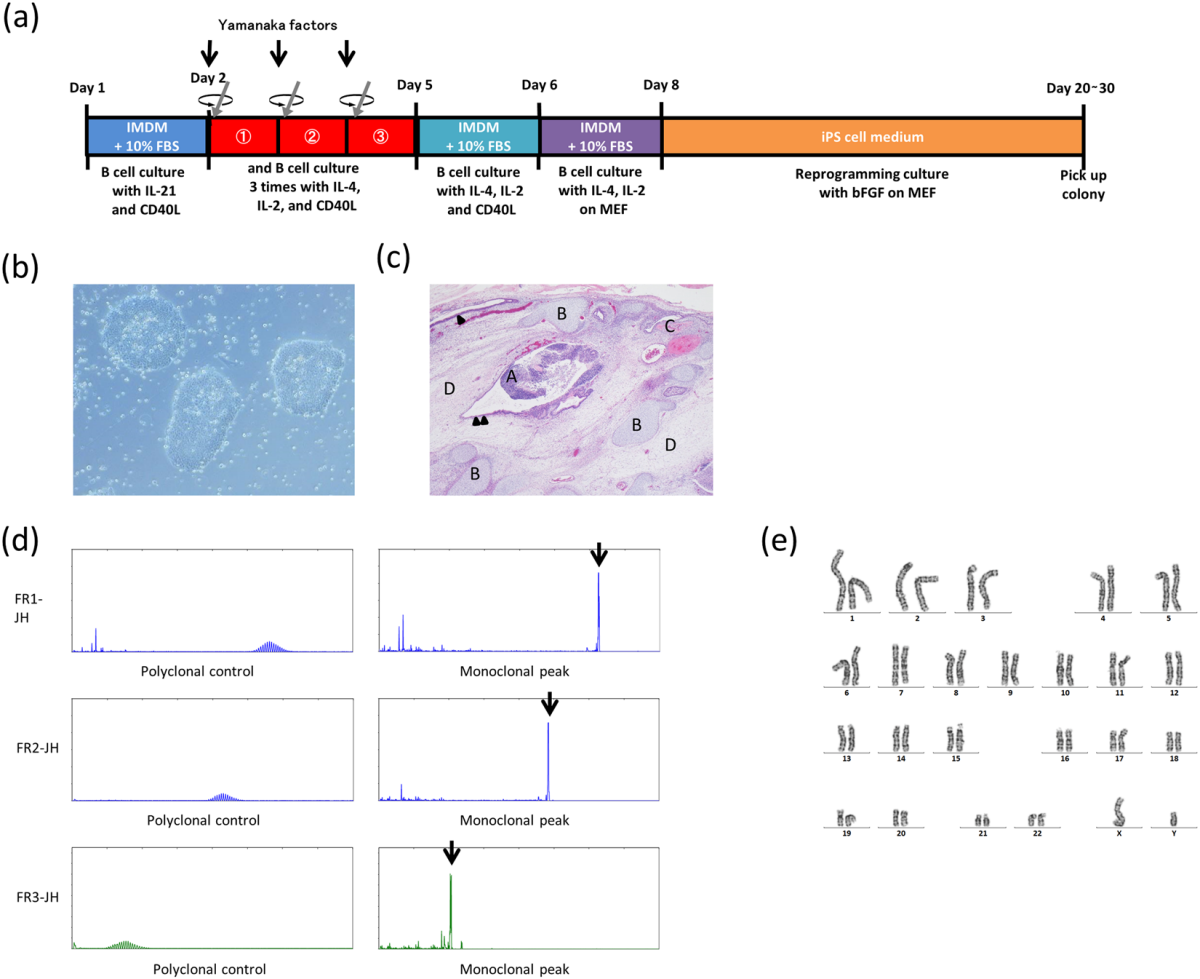
Generation of B cell derived iPSC (BiPSC). (**a**) Time schedule and protocol of BiPSC generation. (**b**) Colony formation of a representative BiPSC13 (x200). (**c**) Teratoma derived from human BiPSC13 (x4). Nine weeks after the injection of BiPSC13, teratomas were dissected from bulging lower abdomen. Hematoxylin and eosin staining of the teratoma. (A) Nerve tissue (ectoderm), (B) Cartilage tissue (mesoderm), (C) Bone tissue (mesoderm), (D) Connective tissue (mesoderm) and endodermal epithelial tissues with a lumen structure (Arrow head), and ectodermal epithelial tissue consisting of ependymal cell-like cells (Double arrow heads). (**d**) Monoclonal VDJ rearrangements of the IgH gene (Arrows) in BiPSC13 detected using PCR. (**e**) Normal G-banding karyotype of BiPSC13.

Normal B cells were purified from the LNs of patients who underwent mediastinum surgical treatment. Cell surface analysis of the LN lymphocytes before the CD19^+^ lymphocytes purification indicated that about 70% of the lymphocytes were T cells and about 20% were B cells (Fig. 1a). In terms of expression of CD27, which is a memory B cell marker, there were two populations within B cells, a CD27^+^ and a CD27^−^ population (Fig. 1a). Over 99% pure B cells were obtained by purification using the CD19-microbeads (Fig. 1b), and subsequently, the Yamanaka factors were transfected into these B cells using a retroviral vector. The processes of cell culture and virus transfection are shown in Fig. 2a. The critical points of these processes are that, after pre-stimulation of the B cells with IL-21 and CD40L, three successive transfections and stimulations were performed, in each of which, after the transfection of highly concentrated Yamanaka factors into the B cells, the cells were centrifuged for 2 h and were then stimulated with IL-4, IL-2, and CD40L for 22 h. Subsequently, the infected B cells were cultured with IL-4, IL-2, and CD40L for 1 day and were then cultured on MEFs with IL-4 and IL-2 for 2 days, followed by continuous culture on MEFs with bEGF and iPSC medium until the occurrence of colony formation. Forty-eight colonies were picked up from 3 × 10^6^ of the purified B cells and IgH gene rearrangements were confirmed in 17 of these colonies. Teratoma formation was examined in 6 of these 17 clones, and was confirmed in 4 of the clones. Based on these data, we established 4 BiPSCs from normal B cells in LN, in which 3 BiPSCs were confirmed to have a normal karyotype and to be negative for EBV infection (data not shown). Although all colonies were not confirmed to be derived from B cells, BiPSC induction efficiency from normal B cells in LN was 0.0016%. A representative BiPSC, that we called BiPSC13, was analyzed in the following studies. The results of PCR showing the IgH gene rearrangement, the colony formation, the teratoma formation, and the karyotype of BiPSC13 are shown in Fig. 2.

**Figure 3:**
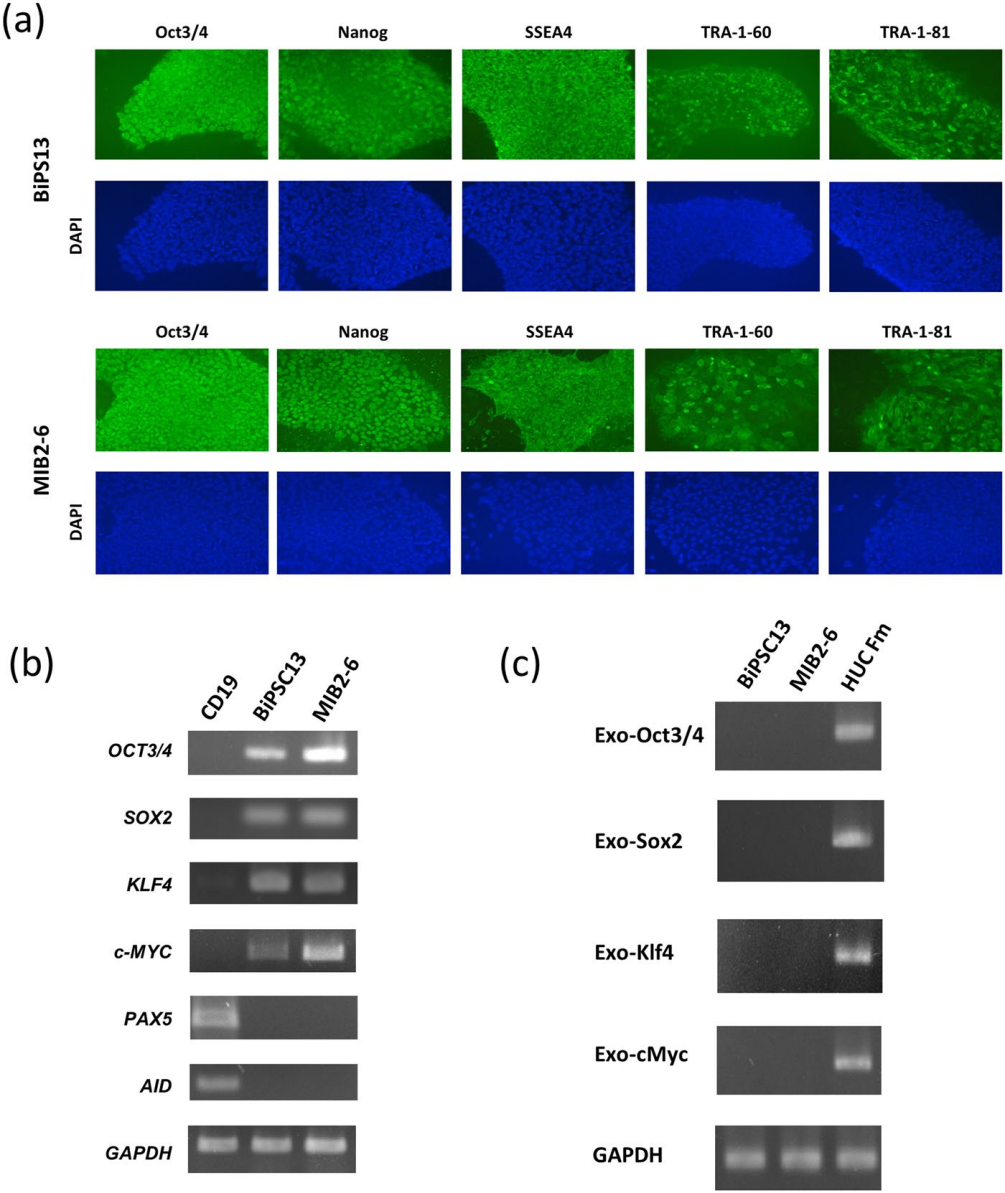
Characterization of the BiPSCs. (**a**) Immunofluorescence staining of BiPSC13 and MIB2-6 for expression of the pluripotent markers Oct3/4, Nanog, SSEA4, TRA-1-60, and TRA-1-81. (**b**) Expression of endogenous *Oct3/4, Sox2, Klf4, cMyc, Pax5, AID*, and *GAPDH* in BiPSCs (BiPSC13, MIB2-6) and normal B cells (CD19) from the lymph node analyzed using RT-PCR. (**c**) RT-PCR analysis of the expression of retrovirus-derived *Oct3/4, Sox2, Klf4*, and *cMyc* in BiPSCs (BiPSC13, MIB2-6). HUC-Fm, human umbilical cord fibroblast cells, (male; RIKEN, Tsukuba, Japan) infected with a retrovirus containing *Oct3/4*, *Sox2*, *Klf4*, *c-Myc*, and *GAPDH* for 5 days were used as the positive control.

In the following studies, the BiPSC cell line MIB2-6 that was established by Inaki *et al*. from normal human peripheral B cells (unpublished cell line, see supplementary data, Supplementary Figures 1 and 2) was analyzed in parallel with BiPSC13. BiPSC13 and MIB2-6 (BiPSCs) were positive for the pluripotent markers Oct3/4, Nanog, SSEA4, TRA-1-60, and TRA-1-81 by immunofluorescence staining (Fig. 3a). RT-PCR analysis showed that BiPSC13 and MIB2-6 expressed endogenous *Oct3/4, Sox2, Klf4*, and *cMyc*, but did not express the B cell marker *Pax5*, or *AID* (Fig. 3b). We also confirmed the loss of the B cell markers CD19, CD20, and CD27 in these cells using flow cytometery (Supplementary Figure 3). Retrovirus-derived *Oct3/4*, *Sox2*, *Klf4*, and *cMYC* were not expressed in these cells as assessed using RT-PCR (Fig. 3c).

### Differentiation of BiPSCs into hematopoietic progenitors and colony-forming assay

**Figure 4:**
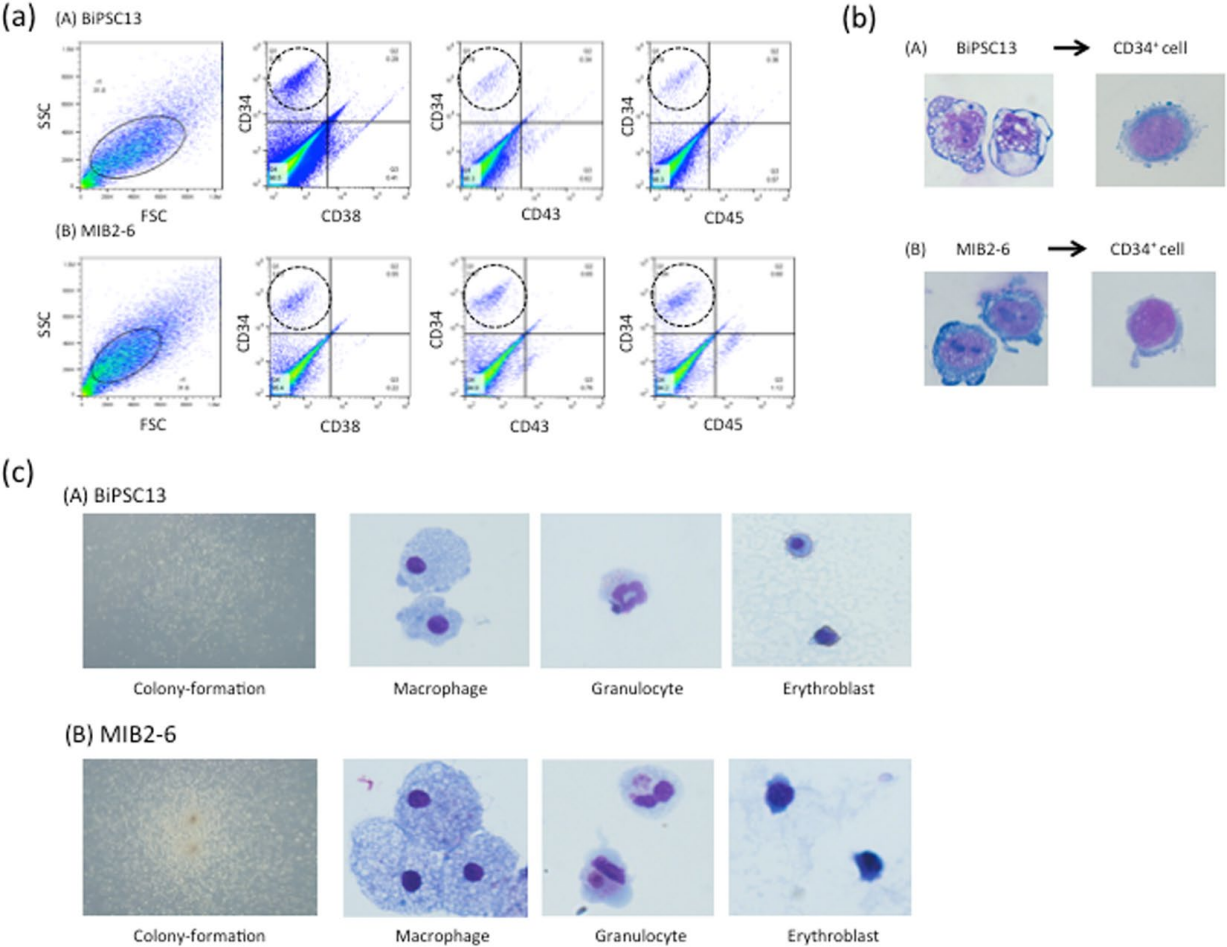
Hematopoietic progenitor cells differentiation of BiPSCs. (**a**) Flow cytometric analysis of the cell phenotype after differentiation of BiPSCs into HPCs. (A) BiPSC13, (B) MIB2-6. The population of CD34-positive cells is surrounded by a dotted line. (**b**) Representative morphology (x1000) and Wright stain of BiPSC13 (A) and MIB2-6 (B) cells before and after differentiation into CD34^+^ cells. (**c**) Morphology of formed colonies (x50) and Wright staining of cytospins picked up from a colony (x1000). (A) BiPSC13, (B) MIB2-6.

In order to confirm the capacity of these two BiPSCs to differentiate into HSCs, we cocultured BiPSC13 and MIB2-6 with C3H10T1/2 cells. On day 14 of culture, the cells were collected and the emergence of HSCs was analyzed using flow cytometry (Fig. 4a). A population of CD34^+^/CD38^−^ cells was detected and sorted. These cells, which were obtained from both BiPSC13 and MIB2-6 cultures, were morphologically similar to HSC cells (Fig. 4b). However, these cells were negative for CD43 and CD45. Since the cells were negative for CD43, which is a marker of HSCs, we next performed a colony-forming assay to confirm that these CD34-positive cells had the capacity to undergo differentiation. Although the typical erythroid colony-forming unit was not detected, colony formations with mixed macrophages and granulocytes were observed (2–3/0.6–3.0 × 10^4^ of CD34-positive cells) and macrophages, granulocytes, and erythroblasts were confirmed in the cells picked-up from those colonies (Fig. 4c). The phenotype of CD34^+^/CD38^−^/CD43^−^/CD45^−^ is similar to that of hematoendothelial cells as Vodyanik proposed previously^24^.

### Establishment of BiPSCs with inducible AID expression, using the doxycycline-controlled (Tet-off) system

**Figure 5:**
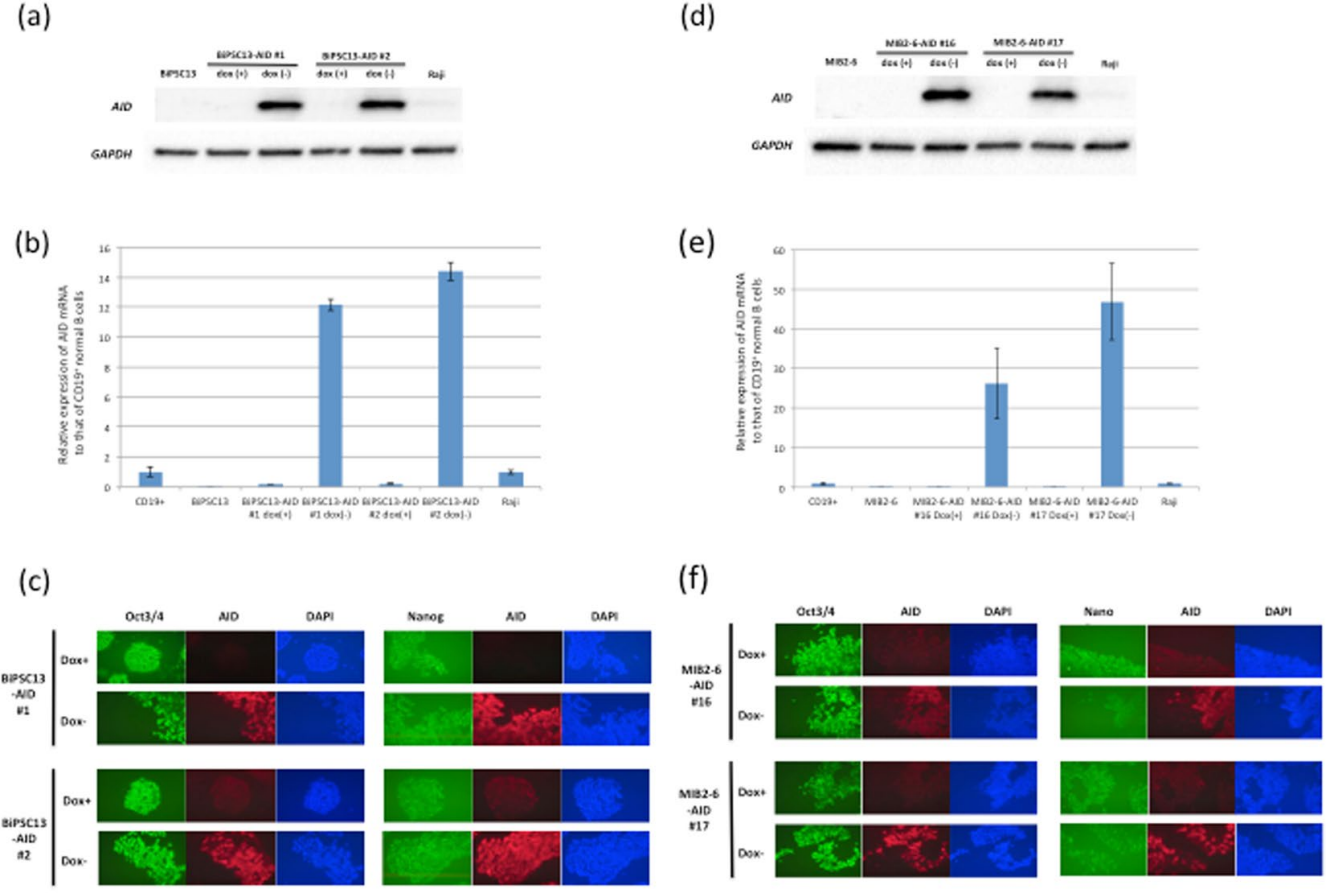
AID expression in BiPSCs induced with the doxycycline-controlled (Tet-off) system. (**a**) Induction of AID expression in the absence of doxycycline was confirmed in two clones (#1 and #2) derived from BiPSC13 by western blotting, and (**b**) qRT-PCR. In (**b**), the numbers on the Y axis are the relative ratio comparing the expression of AID mRNA of each sample standardized by the expression of GAPDH mRNA with that of CD19^+^ normal B cells purified from normal LN using CD19-microbeads. Raji, Burkitt lymphoma cell line. Data were analyzed in triplicates and normalized to glyceraldehyde 3-phosphate dehydrogenase. (**c**) Immunofluorescence analysis of the expression of Oct3/4 and Nanog in BiPSC13-AID (#1 and #2) after the induction of AID expression in the absence of doxycycline. (**d**) Induction of AID expression in the absence of doxycycline in two clones (#16 and #17) derived from MIB2-6 by western blotting, and (**e**) qRT-PCR as described in the legend to (**b**). (**f**) Immunofluorescence analysis of the expression of Oct3/4 and Nanog in MIB-AID (#16 and #17) after the induction of AID expression in the absence of doxycycline.

**Figure 6:**
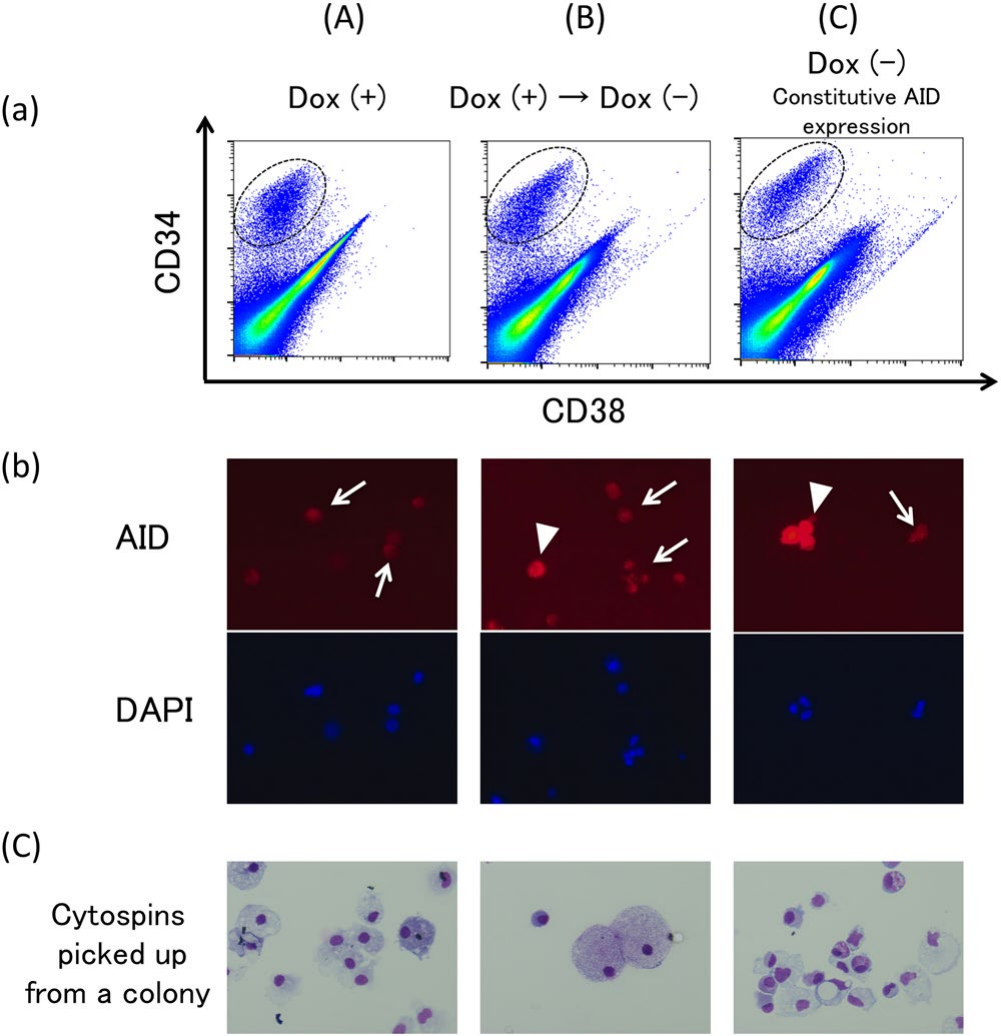
Hematopoietic differentiation from BiPSC13 with AID expression induced using the doxycycline-controlled (Tet-off) system. (**a**) Flow cytometric analysis of the cell phenotype after differentiation of BiPSC13#1-AID into hematopoietic progenitors. The population of CD34-positive cells is surrounded by a dotted line. (A) BiPSC13#1-AID were cultured in the presence of doxycycline, (B) Originally, BiPSC13#1-AID were cultured in the presence of doxycycline to inhibit expression of AID, and subsequently, doxycycline was withdrawn 10 days before sorting of the CD34-positive cells, and (C) BiPSC13#1-AID were cultured in the absence of doxycycline to express AID constitutively. (**b**) Immunofluorescence analysis of the expression of AID in the sorted CD34-positive cells. AID expressed cells were detected in (B) and (C) partially. Arrow heads and arrows indicate AID-positive or –negative cells, respectively. Culture condition of (A), (B), and (C) are as described in (**a**). (**c**) Wright staining of cytospins picked up from a colony (x400). Culture condition of (A), (B), and (C) are as described in (**a**).

The above data confirmed that the two different BiPSCs tested were capable of differentiating into hematopoietic stem cells. We therefore expected that it would be possible to induce chromosomal translocations in B cells if we could establish BiPSCs with induced AID expression and differentiate them into HSCs. Furthermore, we expected that we could differentiate these BiPSCs into B cells in which double stranded breaks (DSBs) of DNA would be induced by AID expression. We established two BiPSCs for each BiPSC13 and MIB2-6 in which AID expression could be induced by doxycycline-controlled (Tet-off) system (BiPSC13-AID (#1 and #2) and MIB2-6-AID (#16 and #17) termed BiPSCs-AID; Fig. 5). Induced expression of AID mRNA peaked at 72 h after the removal of doxycycline and disappeared at 24 h after doxycycline addition (Supplementary Figure 4) and immunofluorescence staining of AID was detected at 2 or 3 days after the removal of doxycycline (Supplementary Figure 5). These BiPSCs-AID cells expressed Oct3/4 and Nanog and after the induction of AID expression (Fig. 5c,f). AID expression had no effect on cell proliferation (Supplementary Figure 6). We then treated two BiPSCs-AID (BiPSC13#1 and MIB2-6#17) as described above to differentiate them into HSCs and removed doxycycline 10 days before sorting of the CD34-positive cells. Both BiPSC13#1 with induced AID expression and BiPSC13#1 without induced AID expression were capable of differentiating into HSCs (Fig. 6). Furthermore, there was no difference between BiPSC13#1 with induced AID expression in the course of differentiation into HSCs and BiPSC13#1 with constitutive AID expression. AID expression in the sorted CD34-positive cells was confirmed by immunostaining (Fig. 6b). Furthermore, we detected macrophages and granulocytes within the CD34-positive population in a colony-formation assay (3–5/1.3–4.0 × 10^4^ of CD34-positive cells) (Fig. 6c). Although the colony-formation assay was not performed due to a small number of CD34-positive cells, both MIB2-6#17 with induced AID expression and MIB2-6#17 without induced AID expression were also capable of differentiating into HSCs (Supplementary Figure 7). Therefore, we succeeded in establishing a BiPSC in which it was possible to induce AID expression using a doxycycline-controlled system, and resulting AID expression did not affect the differentiation of the BiPSC into HSCs.

## Discussion

Initially, we tried to establish iPSCs from B cells purified from lymph nodes using a retroviral vector for the transfection of C/EBPα followed by transfection of Yamanaka factors; however no colony formation indicating the generation of iPSCs was observed. It is possible that, compared with the method of Bueno *et al*. who used a Sendai virus vector^15^, the transfection efficiency of genes using the retroviral vector of our study might be lower. Ultimately, we succeeded in the establishment of BiPSCs from B cells using Inaki’s method (unpublished method; supplemental Materials and Methods). The original B cells obtained from the lymph node biopsy were CD20^+^/CD27^+/−^ (Fig. 1) by cell surface antigen analysis after. Therefore, it is not clear whether the original B cells from which the BiPSCs were derived were memory B cells or not.

Noteworthy points in our method were the activation of B cells with IL-2, IL-4, IL-21, and CD40L, and subsequently, centrifugation of B cells for 2 h onto a retronectin coated plate during the transfection of Yamanaka factors. Furthermore, these procedure were repeated three times. The reprogramming efficiency (iPSC clones/infected cells^15^) of our method appears to be close to the efficiency reported by Bueno.

**Figure 7:**
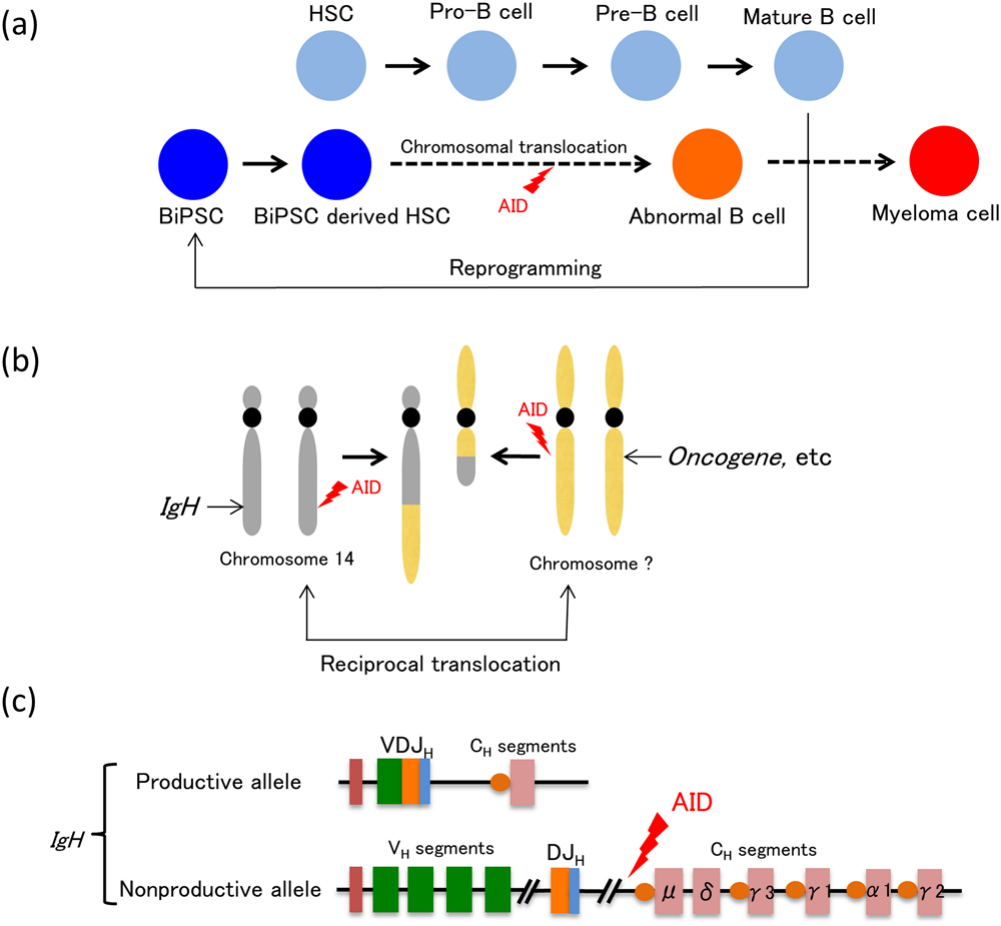
Abnormal B cells as the origin of myeloma cells (hypothesis). When expression of Yamanaka factors is induced for any reason in mature B cells, the cells are reprogrammed and B cell derived iPS cells (BiPSCs) are established (**a**). During the process of redifferentiation of these BiPSCs into hematopoietic stem cells and further into B cells, cleavages occur on chromosome 14 and another chromosome due to AID expression, resulting in the formation of abnormal B cells (**a**) with reciprocal translocation of these chromosomes (**b**). One of the alleles of the IgH gene of these B cells is a productive (functional) allele that had performed VDJ rearrangement as well as class switch recombination and produces the so-called M protein. The other allele, however, is a nonproductive (nonfunctional) allele (**c**), in which case binding to oncogenes, etc. (**b**) due to reciprocal translocation with another chromosome following cleavage between the J and C segments due to AID expression results in myelomagenesis.

The purpose of establishing BiPSCs from normal B cells was to find or make an abnormal B cell that could be considered as an origin of B lymphoid tumors. The BiPSCs of the present study originated from B cells in lymph nodes or peripheral blood and have IgH gene rearrangement. Therefore, these BiPSCs were thought to be established from germinal center (GC) or post-GC B cells. Furthermore, taking into consideration the fact that these BiPSCs have a teratoma-forming ability, if a chromosome brake were to occur either in the allele with IgH gene rearrangement (productive allele) or in another nonproductive allele of the IgH gene, and then a chromosome reciprocal translocation with another chromosome with an oncogene were to happen, the reactivation of BiPSCs to differentiate into B cells might be a cause of B cell neoplasms *in vivo* (Fig. 7).

The chromosomal translocation t(14;18) in follicular lymphoma (FL) and t(11;14) in mantle cell lymphoma (MCL) are thought to occur at the time of VDJ rearrangement of the IgH gene in the process of B cell differentiation. Therefore, the origin of these lymphoma cells is a B cell in the bone marrow (BM) or a B cell in pre-GC. On the other hand, a myeloma cell is thought to originate from a GC or a post-GC B cell and has a productive (functional) allele with IgH gene rearrangement and class switch recombination (CSR), which produces the M-protein, and another nonproductive (nonfunctional) allele of chromosome 14. Usually, the latter allele is thought to reciprocally translocate with other chromosomes in the myeloma cell. Therefore, we hypothesize that the origin of a myeloma cell would be a reprogrammed mature B cell, in which reciprocal chromosome translocation would occur by a double stranded break (DSB) of DNA induced by AID activation (Fig. 7). Indeed, the CD19 antigen on the cell surface and its transcriptional factor, Pax5, are deleted in myeloma cells unlike in other B cell lymphomas^25^. Furthermore, myeloma cells expressing CD33^26^, which is a cell surface marker of granulocytes, or producing amylase^27, 28^ or ammonia^29^ have been reported. The two BiPSCs of our study did not express Pax5 and were negative for CD19 (Fig. 3, Supplementary Figure 3). Those features suggest that myelomagenesis might result from transformation from a mature B cell accompanied by reprogramming of the mature B cell.

Here we reported two BiPSCs that were established from B cells of lymph nodes and peripheral blood, in which AID expression could be induced using the tet-off system. Furthermore, these BiPSCs were able to differentiate into hematopoietic stem cells. Generally, AID is an enzyme that initiates SHM and CSR in B cells. However, we did not detect an increase in DIC formation, which is evidence of DSB of DNA, in the BiPSCs. The DIC analysis was performed at 10 days based on the result that the expression of AID mRNA and protein already peaked at this time after the removal of doxycycline in the tet-off system (Supplementary Figures 4 and 5). Furthermore, there was no significant difference in the number of DIC formed in the BiPSCs with and without constitutive AID expression (data not shown). On the other hand, only 5% of the sorted CD34-positive cells in the experiment of BiPSCs differentiation into HPCs were AID positive cells. Therefore, the capability of inducing AID expression in the tet-off system might differ between BiPSCs and CD34-positive cells.

If we could differentiate these BiPSCs into mature B cells *in vitro* or in a mouse, we could prove the relationship between reprogramming and oncogenesis in mature B cells.

## Materials and Methods

### Ethics Statement

The samples and the medical records used in this study were approved by the Ethics Committee of the Fukushima Medical University School of Medicine (approval number 14999). Written informed consent was obtained from all patients undergoing surgical treatment for analysis of their Lymph node (LN) samples, and the protocols were carried out in accordance with approved guidelines of the Council for International Organizations of Medical Science^30^.

### Phenotype analysis

LN biopsy samples were minced and evaluated by two-color flow cytometry after staining in phosphate-buffered-saline without calcium chloride magnesium chloride (PBS (−)) with the following monoclonal antibodies: anti-CD19-phycoerythrin (PE) (BioLegend, San Diego, CA, USA), anti-CD27-PE (BioLegend), anti-CD3-PE (BioLegend), anti-CD20-fluorescein isothiocyanate (FITC) (BioLegend), and anti-CD38-FITC (BioLegend). BiPSCs were also evaluated by two-color flow cytometry before and after the induction of hematopoietic differentiation using the following monoclonal antibodies: anti-CD19-PE, anti-CD27-PE, anti-CD34-PE (BioLegend), anti-CD20-FITC, anti-CD38-FITC, anti-CD43-FITC (BioLegend), and anti-CD45-FITC (BioLegend). Immunofluorescence of the labeled cell membrane was evaluated using a BD FACSCANTO II flow cytometer (Becton Dickinson, Franklin Lakes, NJ, USA).

### Plasmid construction

The plasmid vectors used in this study were constructed as described in the Materials and Methods in Supplemental Data.

### Establishment of the PG13/OSKMG cell line

The retroviral producing cell line (PG13/OSKMG) was established as described in the Materials and Methods in Supplemental Data.

### Establishment of the 293FT/CD40L cell line and preparation of a highly concentrated CD40L supernatant

These procedures are described in the Materials and Methods in Supplemental Data.

### Generation of BiPSCs and cell culture

Figure 2a BiPSCs were established from normal B cells purified from the LNs of patients who underwent surgical treatment. B cells were purified using magnetic-activated cell sorting CD19 Micro Beads (Miltenyi Biotec, Auburn, CA, USA) according to the manufacturer’s instructions. Approximately 3 × 10^6^ CD19^+^ cells were suspended in 4 ml B cell culture medium (Iscove’s Modified Dulbecco’s Medium (IMDM) (Life Technologies, Funabashi, Japan)/10% fetal bovine serum (FCS) (Equitech-Bio, Kerrville, TX, USA)/PS: Penicillin (50 U/ml) and Streptomycin (50 μg/ml) (Nacalai Tesque, Kyoto, Japan)/L-glutamine (4 mM) (Life Technologies, Funabashi, Japan)) and were cultured after adding IL-21 (25 ng/ml) (PeproTech, Rocky Hill, NJ, USA) with the concentrated supernatant of 293FT/CD40L cells (Supplemental Materials and methods) (20 μl, 1/200 of total volume, equivalent to 2 ml of supernatant/well) in a 6-well plate (IWAKI, Funabashi, Japan) and incubated for 39 h (Day1–2). Activated B cells were collected and transferred into 4 ml of cytokine-free culture medium (2 ml/well) containing protamine sulfate (1 mg/ml) (Wako, Osaka, Japan), and HEPES (20 mM) (SIGMA, Tokyo, Japan) in a 6-well plate coated with Retronectin (recombinant human fibronectin fragment CH-296, 10 μg/cm^2^; TaKaRa, Kusatsu, Japan). Transduction of 40 μl of the concentrated retroviruses (Supplemental materials and methods) containing Oct3/4, Sox2, Klf4, and c-Myc into B cells was performed by spin-infection (2000 × *g*, 2 h). The infected B cells were cultured in 2 ml of new B cell culture medium containing IL-4 (10 ng/ml) (R & D systems, Minneapolis, MN, USA), IL-2 (100 U/ml) (R & D systems), and 20 μl of the concentrated supernatant of 293FT/CD40L cells for 22 h. The above procedure of transduction and culture was performed three times (Day2–5). The infected B cells were collected and cultured in 1 ml of new B cell culture medium containing IL-4 (10 ng/ml), IL-2 (100 U/ml), and 10 μl of the concentrated supernatant of CD40L in a non-coated 6-well plate for 1 day (Day5–6). In parallel, embryonic fibroblast (MEF) feeder cells (CELL BIOLABS, San Diego, CA, USA) were spread on an 100 mm dish (Falcon, Cornig, NY, USA) coated with gelatin (Wako, Osaka, Japan) and were cultured in MEF culture medium (DMEM-high glucose (Nacalai Tesque, Kyoto, Japan)/10% FCS/PS) for 1 day (Day5–6). The next day, the infected B cells were transferred onto the MEF feeder cells and were cultured in new B cell culture medium containing IL-4 (10 ng/ml) and IL-2 (100 U/ml). After two days (Day6–8), the medium was replaced with human iPSC medium (DMEM/F12 (SIGMA) supplemented with 20% Knocout™ Serum Replacement (Thermo Fisher Scientific, Yokohama, Japan), L-glutamine (4 mM) (Thermo Fisher Scientific), MEN Non-Essential Amino Acids Solution (1 mM) (Thermo Fisher Scientific), β-mercaptoethanol (0.1 mM) (Sigma), 1% antibiotic**-**antimycotic (Thermo Fisher Scientific), and basic fibroblast growth factor (10 ng/ml) (bFGF, PeproTech). The human iPSC medium was changed every other day for 6 days (Day8–14). Additionally, sodium butyrate (0.5 mM) (Sigma) was added into the medium until colonies appeared. After culture of the infected B cells for 6 days (Day8–14), the medium was changed to MEF culture medium containing bFGF (10 ng/ml) until colonies were picked up. Established BiPSCs were maintained on a 6-well plate coated with iMatrix-511 (Nippi, Tokyo, Japan) instead of feeder cells in Complete StemFit® AK02N (BiPSC medium) (ReproCELL, Yokohama, Japan). The human iPSC culture medium was changed every day.

### DNA extraction

Genomic DNA was isolated with the DNAiso kit (Takara, Kusatsu, Japan) according to the manufacturer’s instructions. Briefly, the cell pellet was suspended in DNAiso reagent. After removal of the cell debris by centrifugation, the DNA was precipitated by adding ethanol and was recovered by centrifugation at 4,000 × *g*.

### Detection of monoclonal immunoglobulin heavy (IgH) gene rearrangements

PCR analysis of immunoglobuline heavy chain (IgH) gene rearrangements (VH-JH and DH-JH) according to the BIOMED-2 guidelines^31^, using DNA extracted from BiPSCs, was performed in LSI Medience (LSI Medience Corporation, Tokyo, Japan).

### G-banding karyotype analysis

Analysis of the G-banding karyotype was performed in LSI Medience (LSI Medience Corporation, Tokyo, Japan).

### Teratoma formation

BiPS cells were washed with D-PBS (−) (Wako, Osaka, Japan) treated with accutase® (Life Technology) for 5 min at 37 °C, and collected by centrifugation. The cells (5 × 10^7^ cells/ml) were suspended in D-PBS (−) on ice. Subsequently, the cells were injected subcutaneously into the testicle of SCID mice (C.B-17/Icr-scid/scidJcl) (UNITECH, Kashiwa, Japan). Nine weeks after the injection, teratomas were dissected from bulging lower abdomen, and paraffin sections were stained with Hematoxylin and Eosin (HE) for all histological determinations.

### Establishment of BiPSCs with AID expression regulated by the doxycycline-controlled (Tet-off) system

AID cDNA was amplified from pcDNA3.1AID (a kind gift of Dr. Yakushijin, Ehime University School of Medicine)^32^ with KOD plus DNA polymerase using the primers: hAID-BamHI, 5′-TGGGATCCgccaccatggac-3′ and hAID-EcoRI, 5′-CAGAATTCtcaaagtcccaaagtacg-3′. Amplified DNA was digested with BamHI and EcoRI, and cloned into the BamHI/EcoRI sites of pRetroX Tight Pur vector (Clontech) to form pRetroX Tight AID Pur. The DNA sequence of the cloned AID fragment was confirmed. Next, pRetroX Tight AID Pur was transfected into gp293 packaging cells with pMD2.G and the culture supernatant was recovered 3 days later. The supernatant containing packaged retrovirus pseudotyped with VSV-G was added to tTA2^s^ expressing BiPSC13 and MIB2-6 cells. Puromycin resistant cell clones were picked and cultured in the presence of doxycycline (0.2 mg/ml) to prevent AID expression.

### Immunofluorescence staining

BiPSCs were washed with PBS and fixed in 4% paraformaldehyde for 10 min at room temperature (RT). After washing with PBS, the cells were permeabilized with 0.2% Triton X-100 in PBS for 15 min at RT, washed 3 times with PBS, and blocked in PBS-10% goat serum for 30 min. Antibodies (diluted with 1.5% goat sera) against Oct4 (Abcam, Tokyo, Japan), SSEA3 (Abcam), SSEA4 (Abcam), Nanog (Abcam), Tra1-60 (Merck Millipore, Temecula, CA), and Tra1-81 (Merk Millipore) were incubated for 60 min at RT. The cells were washed 3 times with PBS and were then incubated with Alexa Fluor® 488 Goat anti-Mouse IgG (H + L) (Life Technology, Tokyo, Japan), for 45 min at RT. Rabbit anti-AICDA (Abcam) and Alexa Fluor® 594 donkey anti-Rabbit IgG (H + L) (Life Technology) were used to stain AID. Nuclei were counterstained using VECTASHIELD® mounting medium containing 4′,6-diamidino-2-phenylindole (DAPI) (Vector Laboratories, Burlingame, CA, USA).

### Reverse transcription-PCR (RT-PCR) and quantitative RT-PCR (qRT-PCR)

Total RNA was isolated from cells using the RNeasy Mini Kit (Qiagen, Valencia, CA, USA) and first-strand cDNA was synthesized using the First-Strand cDNA Synthesis Kit (Amersham Pharmacia Biotech, Piscataway, NJ, USA) according to the manufacturer’s instructions. The polymerase chain reaction (PCR) was performed using the LifeECO Thermal Cycler (Bioer Technology, Zhejiang, China), and qRT-PCR was performed using Faster Essential DNA Green (Roche Diagnostics, Mannheim, Germany) and the Light Cycler® Nano (Roche). The primers used for RT-PCR and qRT-PCR are listed in the Supplementary table and are mainly quoted from the study performed of Takahashi *et al*.^1^.

### Western blot analysis

Cells were lysed in lysis buffer (0.5% NP-40, 1% TritonX-100, 150 mM NaCl, and 1 mM EDTA in 20 mM Tris, pH 7.5) in the presence of a protease (Nakarai Tesque, Kyoto, Japan) and a PhosSTOP phosphatase (Roche Diagnosis, Tokyo, Japan) inhibitor cocktail. After 30 min on ice, the lysates were centrifuged at 14,000 rpm for 30 min at 4 °C and the supernatants were collected. Protein samples (70 µg aliquots) were separated in a Mini-PROTEAN TGX precast Gel (BIO-RAD, Hercules, CA, USA) and transferred to a nitrocellulose membrane (BIO-RAD). The membrane was immunoblotted with the indicated antibodies, and the signal was visualized using enhanced chemiluminescence (ECL) Western blotting detection reagents (BIO-RAD) and detected with the ChemDoc XRS+ Imaging System (BIO-RAD). The primary antibodies used included anti-AICDA (Abcam, Tokyo, Japan) and anti-GAPDH (Abcam) and the second antibody was goat anti-Rabbit IgG-HRP (Santa Cruz Biotechnology, Dallas, TX, USA).

### Hematopoietic differentiation of BiPSC (Co-culture with C3H10T1/2)

For BiPSC differentiation, we modified a previously described methods^33^. The mouse C3H10T1/2 cell line was purchased from the RIKEN BioResource Center (Tsukuba, Japan) and was plated onto 10-cm dishes in Eagle’s basal medium (Thermo Fisher Scientific) supplemented with 10% FBS (Equitech-Bio, Kerrville, TX, USA), 1% PS, and 1% GlutaMAX^TM^ (Life Technologies, Funabashi, Japan) (C3H10T1/2 culture medium), and was cultured until about 80% confluent. BiPSCs were cultured on a matrigel-coated (Corning, Tokyo, Japan) 6-well plate in Complete StemFit^®^ AK02N for 5–7 days. Subsequently, BiPSCs were treated with DISPASE^®^ II (Wako, Osaka, Japan) for 10 min, and were then picked up as small clumps equivalent to 1 × 10^5^ cells and added to C3H10T1/2 cultures in IMDM (ThermoFisher Scientific) supplemented with 10% FBS, 100 µM monothioglycerol (MTG; Sigma, Tokyo, Japan), 1% PS, 50 mg/ml ascorbic acid, 1% GlutaMAX^TM^, ITS (Sigma), and 20 μg/ml human vascular endothelial growth factor (VGEF) (Peprotech, Rocky Hill, NJ, USA). The BiPSC/C3H10T1/2 cocultures were incubated at 37 °C under normoxic conditions and 5% CO_2_. The medium was replaced with new medium on days 3, 6, 9, and 13. On day 14, BiPSCs were suspended into a single cell strainer and used for phenotype analysis. Concurrently, CD34^+^/CD38^−^ cells were sorted using a cell sorter (S3e^TM^ Cell Sorter; BIO-RAD, Tokyo, Japan), and the collected cells were used for the colony-forming assay or were stained using the Diff-Quick stain™ (Funakoshi, Tokyo, Japan) after cytospin centrifugation.

### Colony-forming assay

CD34^+^ cells derived from BiPSCs obtained following C3H10T1/2 co-culture were sorted using flow cytometry and were then used in colony-forming assays using Methocult H4435 (Stem Cell Technologies, Vancouver, BC Canada) in triplicates. The types of colonies formed were assessed around day 14.

## Electronic supplementary material

Supplementary information
