# Supplementary material for: Establishment of induced pluripotent stem cells from normal B cells and inducing AID expression in their differentiation into hematopoietic progenitor cells
Source: Sci Rep. 2017 May 10;7:1659. doi: 10.1038/s41598-017-01627-1 (PMC5431994; doi:10.1038/s41598-017-01627-1)
Supplement: Supplementary file 1 — Supplementary information [file 41598_2017_1627_MOESM1_ESM.pdf]

Supplemental Information

Establishment of induced pluripotent stem cells from normal B cells and inducing AID expression in their differentiation into hematopoietic progenitor cells

Fumihiko Kawamura[1], Makoto Inaki[2], Atsushi Katafuchi[1], Yu Abe[1], Naohiro Tsuyama[1], Yumiko Kurosu[1], Aki Yanagi[1], Mitsunori Higuchi[3], Satoshi Muto[3], Takumi Yamaura[3], Hiroyuki Suzuki[3], Hideyoshi Noji[4], Shinichi Suzuki[5], Mitsuaki A Yoshida[6], Megumi Sasatani[7], Kenji Kamiya[7], Masafumi Onodera[2], Akira Sakai[1]

[1]Dept. of Radiation Life Sciences, Fukushima Medical University School of Medicine, Fukushima, Japan
[2]Dept. of Genetics, National Research Institute for Child Health and Development, Tokyo, Japan
[3]Dept. of Regenerative Surgery, Fukushima Medical University School of Medicine, Fukushima, Japan
[4]Dept. of Medical Oncology, Fukushima Medical University School of Medicine, Fukushima, Japan
[5]Dept. of Thyroid and Endocrinology, Fukushima Medical University School of Medicine, Fukushima, Japan
[6]Dept. of Radiation Biology, Institute of Radiation Emergency Medicine, Hirosaki University, Hirosaki, Japan
[7]Dept. of Experimental Oncology, Research Institute for Radiation Biology and Medicine, Hiroshima University, Hiroshima, Japan

Correspondence:
Akira Sakai, M.D., Ph.D.
Dept. of Radiation Life Sciences
Fukushima Medical University School of Medicine
1 Hikarigaoka, Fukushima, 960-1295 Japan
TEL: +81-24-547-1703
FAX: +81-24-547-1704
E-mail: sakira@fmu.ac.jp

Materials and Methods

Plasmid construction

The retroviral vector GCsap (myeloproliferative sarcoma virus (MPSV)) was generated by deleting the *NcoI* site in GCsap (MPSV).[1] The open reading frames of human *Oct3/4*, *Sox2*, *Klf4*, and *c-Myc* were amplified from pMXs-h*Oct3/4*, pMXs-h*Sox2*, pMXs-h*Klf4*, and pMXs-h*c-Myc*, retrospectively, using RT-PCR.[2] PCR products were digested with *NotI* and *BamHI* and cloned into GCsap (MPSV).

The lentiviral vector pRRL sin hEF1a-h*CD40L*-IRES-KO-cPPT was constructed via the following procedures: the hEF1a promoter was amplified by genomic-PCR using the primers 5’- AACGCTAGCGGCTCCGGTGCCCGTCA-3’ (Forward primer with an *Nhe*I site) and 5’- GACGATATCGAATTCGGATCCTCACGACACCTGAAATGGAA-3’ (Reverse primer with a *Bam*HI site and an *Eco*RI site). The genomic DNA of Jurkat was used as the PCR template. The PCR products were digested with *Eco*RI and phosphorylated with T4 polynucleotide kinase (NEB, Tokyo, Japan). The DNA fragments were then ligated into pRRL sin hCMV-MCS-cPPT digested with *Hpa*I and *Eco*RI to generate pRRL sin hEF1a-MCS. The ligated plasmids were transformed into the Stbl3 strain of *Escherichia coli* (Life Technologies,

Funabashi, Japan).

The cPPT sequence was amplified by PCR using the primers 5’-CTAGTCTAGATTTTAAAAGAAAAGGGGGGATTG -3’ (Forward primer with an *Xba*I site) and 5’- GGAATTCCTAGCTAGCAAAATTTTGAATTTTTGTAATTTGTTT -3’ (Reverse primer with an *Nhe*I site). pRRL sin hCMV-MCS-cPPT was used as the PCR template. The PCR products were digested with *Xba*I and *Nhe*I. The DNA fragments were then ligated into pRRL sin hEF1a-MCS digested with NheI to generate pRRL sin hEF1a-MCS-cPPT.

The IRES-Kusabira-Orange (IRES-KO) sequence was amplified by PCR using the primers: 5’-CCCTCAAAGTAGACGGCATCGC—3’ (Forward primer) and 5’-GGATATCTTAGCAGTGGGCCACGGCGT—3’ (Reverse primer with an *Eco*RV site). A retroviral vector pGCDN-IRES-KO was used as the PCR template. The PCR products were digested with *Eco*RI and *Eco*RV. The DNA fragments were then ligated into pRRL sin hEF1a-MCS-cPPT digested with *Eco*RI and *Eco*RV to generate pRRL sin hEF1a-MCS-IRES-KO-cPPT.

To modify the multi-cloning site (MCS), pRRL sin hEF1a-MCS-IRES-KO-cPPT was digested with *Bam*HI and *Eco*RI, and treated with bacterial alkaline phosphatase (TaKaRa,

Kusatsu, Japan). Subsequently, 5 µl of each of the synthesized sense and antisense oligonucleotides (100 µM each) were mixed with 40 µl of 150 mM NaCl, denatured at 95 °C for 3 min and annealed by cooling to 37 °C for 2 h. The following oligonucleotides were used: 5’- GATCGGAATTCTCTAGAGGATCCC-3’ (sense oligonucleotides with an *Eco*RI site, an *Xba*I site and a *Bam*HI site) and 5’- AATTGGGATCCTCTAGAGAATTCC-3’ (antisense oligonucleotides). The annealed oligonucleotides were phosphorylated by T4 polynucleotide kinase (Takara) and ligated into the digested pRRL sin hEF1a-MCS-IRES-KO-cPPT to generate pRRL sin hEF1a-MCS2-IRES-KO-cPPT.

Human *CD40L* (h*CD40L*) was amplified by RT-PCR using the primers: 5’-GCTCTAGACACCATGATCGAAACATACAACCAAAC-3’ (Forward primer with an *Xba*I site) and 5’- CGGGATCCTCAGAGTTTGAGTAAGCCAAAG-3’ (Reverse primer with a *Bam*HI site). The cDNA of human activated T cells was used as the RT-PCR template. The PCR products were digested with *Xba*I and *Bam*HI. The DNA fragments were then ligated into pRRL sin hEF1a-MCS2-IRES-KO cPPT to generate pRRL sin hEF1a-h*CD40L*-IRES-KO-cPPT.

pEF-1α Tet-Off-IRES neo was constructed as follows. The tTA2^{s}-IRES neo DNA fragment was PCR amplified using the pRetroX-Tet-Off Advanced vector as a template

(Clontech, Mountain View, CA, USA), KOD plus DNA polymerase (TOYOBO, Osaka, Japan) and the primers: tTA IRES neo-XbaI/F2, 5’-ACTCTAGAACCATGTCAAGACTGGAC-3’ and tTA IRES neo-XbaI/R2, 5’-CCTCTAGATGGGTGGTGAGCAGCTC-3’. The DNA amplimer was digested with XbaI and cloned into the XbaI site of pEF-BOS. pEF-1α Tet-Off-IRES neo was then transfected into BiPSC-13 and MIB2-6 cells using ViaFect (Promega, Madison, WI, USA) according to the manufacturer’s instructions. G418 resistant cells of both cell lines were isolated and used for further doxycycline-controlled activation-induced cytidine deaminase (AID) expression.

Establishment of the PG13/OSKMG cell line

The Retroviral producing cell line (PG13/OSKMG) was established as follows: First, the retroviral plasmid described above or GCsapEGFP (MPSV) [1] was cotransfected with the 10A1 env expression plasmid (pCL-10A1) into 293gp cells by CaPO4 coprecipitation. The 293gp cells were cultured in DMEM-high glucose (Nacalai Tesque, Kyoto, Japan) supplemented with 10% FBS (Equitech-Bio, Kerrville, TX, USA), 1 mM sodium pyruvate (Thermo Fisher Scientific, Yokohama, Japan), 50 U/ml Penicillin and 50 μg/ml Streptomycin (Nacalai Tesque) at 37ºC in 10% CO_2. The supernatant from the transfected 293gp cells was harvested and filtered (0.45 μm pore-size, Millipore, Merck Millipore, Darmstadt, Germany).

The filtered supernatant was concentrated by PEG precipitation (see below under “Concentration of PG13 retrovirus”) [3] and resuspended in serum-free DMEM-high glucose medium. The concentrated retrovirus was used to transduce PG13 cells to express the GaLV-envelope. The PG13 cells were cultured in DMEM-high glucose supplemented with 10% FBS, 50 U/ml Penicillin and 50 μg/ml Streptomycin (D10 medium) at 37ºC in 10%CO_2. Transduction was performed three times, each time by spin-infection (1000×g, 1 h) in the presence of 8 μg/ml polybrene (Nacalai Tesque, Kyoto, Japan) and 20 mM HEPES (Sigma, Tokyo, Japan). After transduction, GFP-positive PG13 cells were sorted using a BD FACS Aria (BD Biosciences, Tokyo, Japan) into 96-well plates. Highly-proliferative cells were amplified and harvested. Proviral cDNA expression was confirmed by RT-PCR. Total RNA was extracted using RNeasy (Qiagen, Tokyo, japan), and the cDNA was synthesized using the Transcriptor High Fidelity cDNA Synthesis Kit (Roche, Tokyo, Japan). PCR was performed with the ExTaq Hot Start Version (Takara). All reactions were carried out with 25 ng cDNA (total RNA equivalent) and 0.5 U of ExTaq Hot Start Version (Takara) in a volume of 20 μl. The final concentrations of the other reactants were: 1x ExTaq Buffer, 0.2 mM each dNTP, 5% DMSO and 0.5 μM of each primer (forward primer 5´-CCCTCAAAGTAGACGGCATCGC-3´ and

reverse primer 5´- TACAGGTGGGGTCTTTCATTCCC-3´). The PCR cycling conditions were as follows: initial denaturation at 94ºC for 2 min followed by 40 cycles of 94ºC for 20 s, 72ºC for 90 s, and a final extension at 72ºC for 5 min. Amplification was performed using a PCR Thermal Cycler Dice (Takara). The PCR products were analyzed by 1.5% agarose gel electrophoresis in TBE buffer. In this manner, PG13/OSKMG cell lines that have all proviral DNAs (*Oct3/4, Sox2, Klf4, c-Myc* and *GFP*) were established.

Concentration of PG13 retrovirus

Established PG13/OSKMG cells were cultured in DMEM-high glucose supplemented with 20% FBS, 50 U/ml Penicillin and 50 μg/ml Streptomycin (D20 medium) at 37ºC in 10% CO_2. PG13/OSKMG cells were seeded in 15 cm dishes at a density of 6.75×10^6 cells/dish. After 3 days of culture, the medium was replaced with fresh medium (D10 medium) for virus production. After 12 h of incubation, the supernatant was harvested and filtered. The supernatant was incubated on ice for 30 min. The chilled supernatant was diluted three times with 4×PEG solution (32% PEG6000 (TOHO Chemical Industry, Tokyo, Japan)/400 mM NaCl/40 mM HEPES, pH7.4) and incubated at 4ºC for 3 h. After this incubation, the

supernatant was centrifuged at 2000×g for 30 min. The supernatant was discarded and the pellet was centrifuged at 2000×g for 5 min again to remove residual supernatant. The final supernatant was removed, and the pellet was resuspended in 1/100th of the original volume of DMEM and stored at -80ºC.

Establishment of the 293FT/CD40L cell line and preparation of a highly concentrated CD40L supernatant

Self-inactivating lentiviral vectors were produced by transient transfection of 293FT cells with the vector plasmid pRRLsin hEF1a-CD40L-IRES Kusabira Orange cPPT, the lentiviral packaging constructs pRSV/REV and pMDLG/pRRE, and the vesicular stomatitis virus glycoprotein (VSV-G)-expressing plasmid pcDNA3-VSVG using the calcium phosphate method. 293FT cells were transduced with concentrated lentiviruses in the presence of 8 μg/ml polybrene. After 5 days, Kusabira Orange-positive cells were sorted using a fluorescence-activated cell sorter and were inoculated into a 96 well plate coated with poly-L-lysine (Cellvis, Mountain View, CA, USA).

Established 293FT/CD40L-IRES-KO cells were cultured in DMEM-high glucose

supplemented with 10% FBS, 50 U/ml Penicillin and 50 μg/ml Streptomycin (D10 medium) at 37ºC in 10%CO_2. 293FT/CD40L-IRES-KO cells were seeded in 15 cm dishes at a density of 6.75×10^6 cells/dish. After 3 days of culture, the medium was replaced with fresh medium (D10 medium) for virus production. After 24 h of incubation, the supernatant was harvested and filtered. The supernatant was incubated on ice for 30 min. The chilled supernatant was diluted three times with 4×PEG solution (32% PEG6000/ 400 mM NaCl/ 40 mM HEPES, pH7.4) and incubated at 4ºC for 3 h. After this incubation, the supernatant was centrifuged at 2000×*g* for 30 min. The supernatant was discarded and the pellet was centrifuged at 2000×*g* for 5 min again to remove residual supernatant. The final supernatant was removed, and the pellet was resuspended in 1/100th of the original volume of the B cell medium (see below) and stored at -80ºC.

Generation of the BiPSC, MIB2-6, from peripheral B cells

Peripheral blood (30 ml) was obtained from healthy donors and mononuclear (MNC) cells were separated by density-gradient centrifugation using Ficoll-Paque PLUS (GE Healthcare, Tokyo, Japan). MNCs were cultured in Iscove's Modified Dulbecco's Medium

(IMDM) (Life Technologies, Funabashi, Japan) supplemented with 10% fetal bovine serum (FBS) (Equitech-Bio, Kerrville, TX, USA)/PS: Penicillin (50 U/ml) and Streptomycin (50 μg/ml) (Nacalai Tesque, Kyoto, Japan)/L-glutamine (4 mM) (Life Technologies, Funabashi, Japan) (B cell medium) in the presence of IL-4 (4 ng/ml, Peprotech) and cyclosporine A (0.7μg/ml) (Tokyo Chemical Industry, Tokyo, Japan). B cells were purified using magnetic-activated cell sorting CD19 Micro Beads (Miltenyi Biotec, Auburn, CA, USA) according to the manufacturer's instructions. The procedure for the generation of BiPSC after the purification of B cells to generate BiPSC is described in the Materials and Methods section of manuscript. In this way, Inaki et al. established the B cell derived induced pluripotent stem cells (iPSCs), MIB2-6.

Dicentric chromosome assay (DCA)

Two different BiPSC cultures in which AID expression was controlled by the Tet-Off system were cultured in BiPSC medium with doxycycline in 6-well plates. After two rounds of washing with PBS and incubation in BiPSC medium for 2 h, duplicate BiPSC cultures were then incubated for 10 days in BiPSC medium without doxycycline.

Colcemid (final concentration, 0.05 μg/ml; Life Technologies) was added 4 h before cell harvesting. BiPSCs were dissociated into single cells by treatment with Accumax (Funakoshi, Tokyo, Japan) and a cell strainer, and then cell harvesting and Giemsa staining was performed according to our previously reported methods.[4]

Cell proliferation assay

The Cell Counting Kit-8 (called the 'WST-8 assay' in this paper) (Dojindo Laboratories, Kumamoto, Japan) was used for the assay of cell proliferation, according to the manufacturer's instructions. Cells were seeded at a density of 1.0×10^5/ml, in a final volume of 100 μl in 96-well flat-bottom plates (CORNING, Corning, NY, USA) in triplicate. The plates were incubated at 37°C in a 5% CO_2 incubator for 1 to 7 days. At the end of the incubation, 10 μl of the WST-8 reagent was added to each well and the plates were incubated for 4 h. Absorbance for 450 nm was then measured using a plate reader (Multiskan FC; Thermo Fisher Scientific, Tokyo, Japan).

Analysis of EBV infection

PCR analysis of the DNA extracted from the BiPSCs was performed with SL18 and SL19 primers to the tandem repeat region of the gene for latent membrane 1 (LMP-1) to detect EBV infection as previously reported.[5]

References

1. Kaneko, S., Onodera, M., Fujiki, Y., Nagasawa, T. & Nakauchi, H. Simplified retroviral vector gcsap with murine stem cell virus long terminal repeat allows high and continued expression of enhanced green fluorescent protein by human hematopoietic progenitors engrafted in nonobese diabetic/severe combined immunodeficient mice. *Hum Gene Ther*. 12, 35-44 (2001).
2. Takahashi, K. *et al*. Induction of pluripotent stem cells from adult human fibroblasts by defined factors. *Cell*. 131, 861-72 (2007).
3. Inaki, M., Kato, D., Utsugi, T., Onoda, F., Hanaoka, F. & Murakami, Y. Genetic analyses using a mouse cell cycle mutant identifies magoh as a novel gene involved in Cdk regulation. *Genes Cells*. 16, 166-78 (2011).
4. Abe, Y. *et al*. Increase in dicentric chromosome formation after a single CT scan in adults. *Sci Rep*. 5, 13882 (2015).
5. Shibata, D., Weiss, L. M., Nathwani, B. N, Brynes, R. K. & Levine, A. M. Epstein-Barr virus in benign lymph node biopsies from individuals infected with the human immunodeficiency virus is associated with concurrent or subsequent development of non-Hodgkin's lymphoma. *Blood*. 77, 1527-33 (1991).

Legends

Table S1. Primer sequences.

Figure S1. Characterization of the B cell derived iPSC, MIB2-6.

(a) Colony-formation of MIB2-6.

(b) Normal G-banding karyotype of MIB2-6.

(c) Hematoxylin-eosin stained teratoma derived from MIB2-6.

Figure S2. Detection of monoclonal immunoglobulin heavy (IgH) gene rearrangements in MIB2-6 cells.

Monoclonal VDJ rearrangements of the IgH gene (arrows) were detected in MIB2-6 using PCR.

Figure S3. Phenotype analysis of BiPSCs.

The cell phenotypes of (a) BiPSC13 and (b) MIB2-6 were analyzed using flow cytometry and the indicated antibodies.

Figure S4. qRT-PCR analysis of AID expression in MIB2-6 induced by the doxycycline-controlled (Tet-off) system.

AID expression in MIB2-6#17 in the absence (a) and the presence (b) of doxycycline was measured using qRT-PCR. The numbers on the Y axis are the expression of AID mRNA standardized by the expression of GAPDH relative to the expression of AID mRNA of $CD19^+$ normal B cells. Data were analyzed in triplicates and normalized to glyceraldehyde 3-phosphate dehydrogenase.

Figure S5. AID expression depending on the number of days after removeing doxycycline by immunofluorescence staining.

Expression of AID was detected in BiPSC13#1 at 2 days (a) and in MIB2-6#17 at 3 days, respectively.

Figure S6. Effect of the absence or presence of doxycycline on cell proliferation.

The cell proliferation of (a) BiPSC13#2 and (b) MIB2-6#17 was measured in the presence

(white circles) or absence (black circles) of doxycycline. Results are expressed as mean ± SD of triplicate experiments.

Figure S7. Hematopoietic differentiation from MIB2-6 with AID expression induced using the doxycycline-controlled (Tet-off) system.

(a) Flow cytometric analysis of the cell phenotype after differentiation of MIB2-6#17-AID into HSCs. The population of CD34-positive cells is surrounded by a dotted line. (A) MIB2-6#17-AID were cultured in the presence of doxycycline, (B) MIB2-6#17-AID were cultured in the presence of doxycycline and then doxycycline was withdrawn 10 days before sorting of the CD34-positive cells, and (C) MIB2-6#17-AID were cultured in the absence of doxycycline to express AID constitutively.

(b) Immunofluorescence analysis of the expression of AID in the sorted CD34-positive cells. AID expressed cells were detected in (B) and (C). Arrow heads and arrows indicate AID-positive or –negative cells, respectively. Culture condition of (A), (B), and (C) are as described in (a).

Table S1. Primer Sequences

Primer (Exo from plasmid vector)	Sequence (5' to 3')	Applications
hOCT3/4-S944	CCC CAG GGC CCC ATT TTG GTA CC	OCT3/4 Tg genomic and RT-PCR
hSOX2-S691	GGC ACC CCT GGC ATG GCT CTT GGC TC	SOX2 Tg genomic and RT-PCR
hKLF4-S1128	ACG ATC GTG GCC CCG GAA AAG GAC C	Tg genomic and RT-PCR
hMYC-S1011	CAA CAA CCG AAA ATG CAC CAG CCC CAG	c-MYC Tg genomic and RT-PCR
pGCsap-AS1	GGG TCT TTC ATT CCC CCC TTT TTC TGG AGA C	Tg genomic and RT-PCR
**Primer (Endogenous RNA)**	**Sequence (5' to 3')**	**Applications**
GAPDH-F	GGT GAA GGT CGG AGT CAA CG	Endo GAPDH RT-PCR
GAPDH-R	AAT TTG CCA TGG GTG GAA TC	Endo GAPDH RT-PCR
hOCT3/4-S1165	GAC AGG GGG AGG GGA GGA GCT AGG	Endo OCT3/4 RT-PCR
hOCT3/4-AS1283	CTT CCC TCC AAC CAG TTG CCC CAA AC	Endo OCT3/4 RT-PCR
hSOX2-S1430	GGG AAA TGG GAG GGG TGC AAA AGA GG	Endo SOX2 RT-PCR
hSOX2-AS1555	TTG CGT GAG TGT GGA TGG GAT TGG TG	Endo SOX2 RT-PCR
hKlf4-sk F	CCC ACA CAG GTG AGA AAC CT	Endo Klf4 RT-PCR
hKlf4-sk R	ATG TGT AAG GCG AGG TGG TC	Endo Klf4 RT-PCR
hMYC-S253	GCG TCC TGG GAA GGG AGA TCC GGA GC	Endo c-MYC RT-PCR
hMYC-AS555	TTG AGG GGC ATC GTC GCG GGA GGC TG	Endo c-MYC RT-PCR
AIDrtPCR-F	AAA ATG TCC GCT GGG CTA AG	Endo AICDA RT-PCR
AIDrtPCR-R	AGG TCC CAG TCC GAG ATG TAG	Endo AICDA RT-PCR
Pax-S	AAT GAC ACC GTG CCT AGC GT	Endo PAX RT-PCR
Pax-AS	GGT GGT GAA GAT GTC TGA GT	Endo PAX RT-PCR

Figure S1

(a)

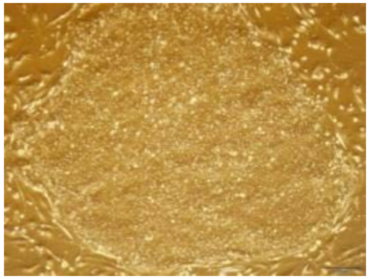

(b)

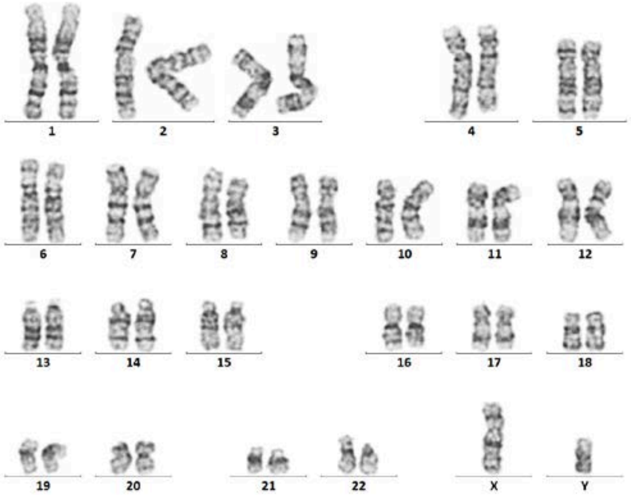

(c)

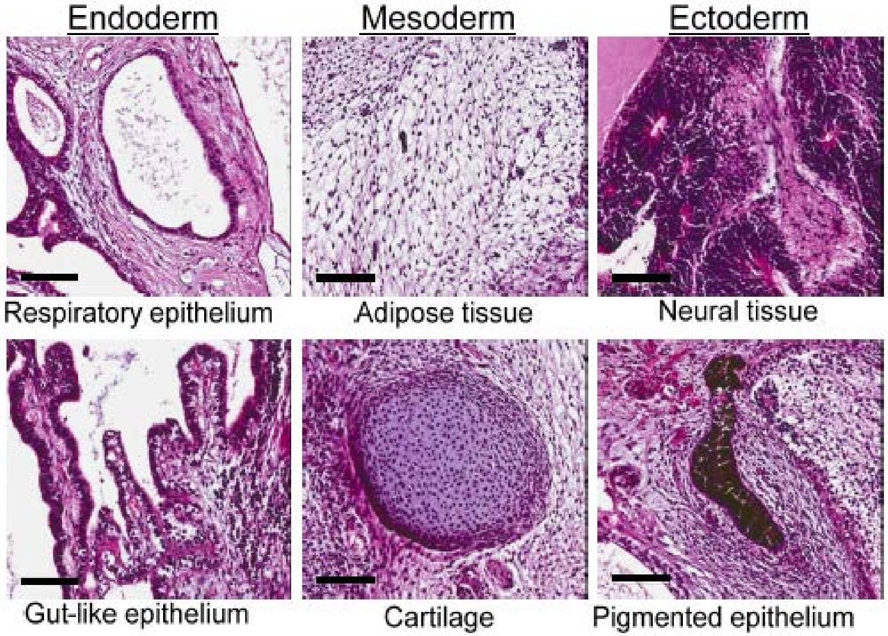

Figure S2

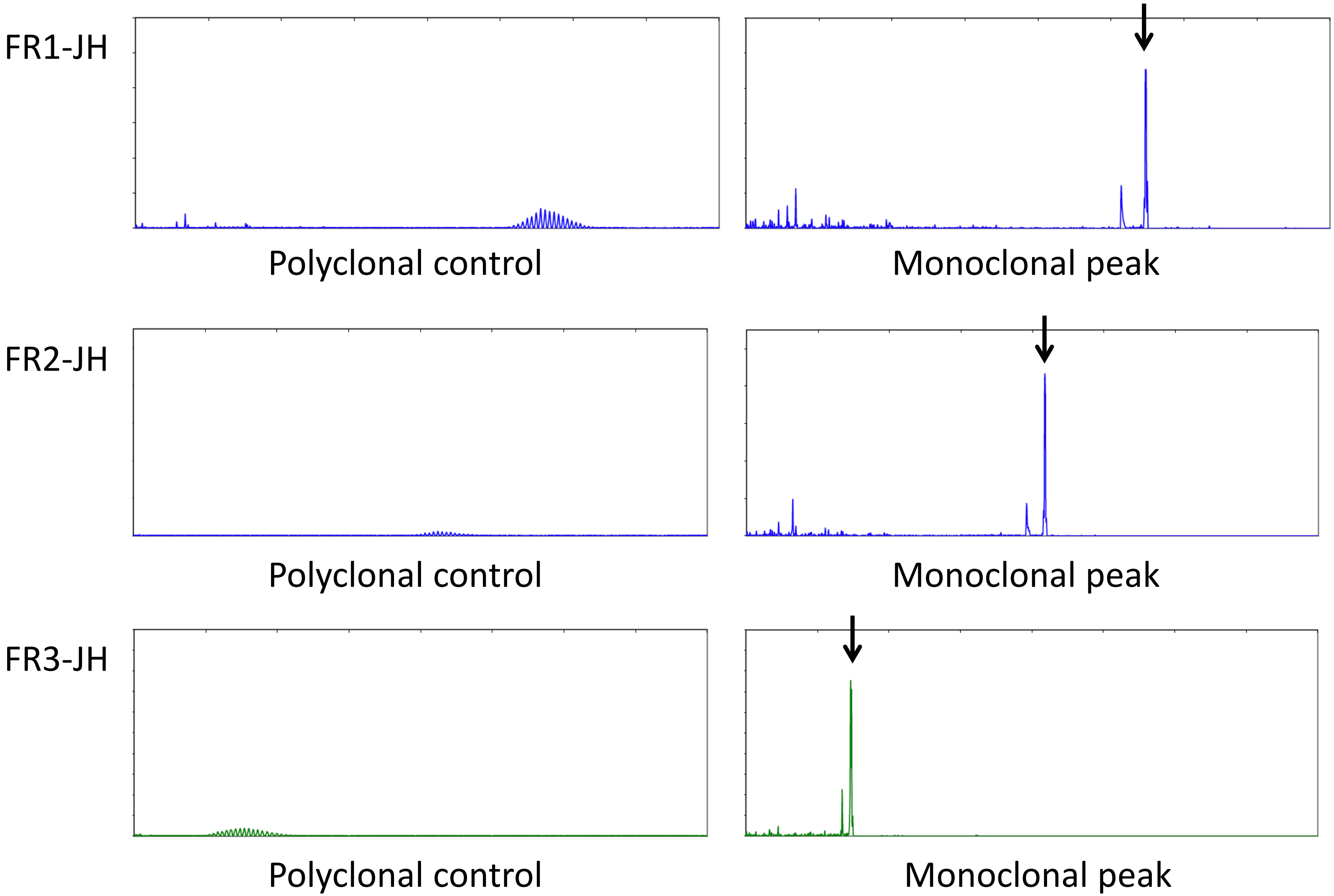
FR1-JH
Polyclonal control
Monoclonal peak
FR2-JH
Polyclonal control
Monoclonal peak
FR3-JH
Polyclonal control
Monoclonal peak

Figure S3

(a)

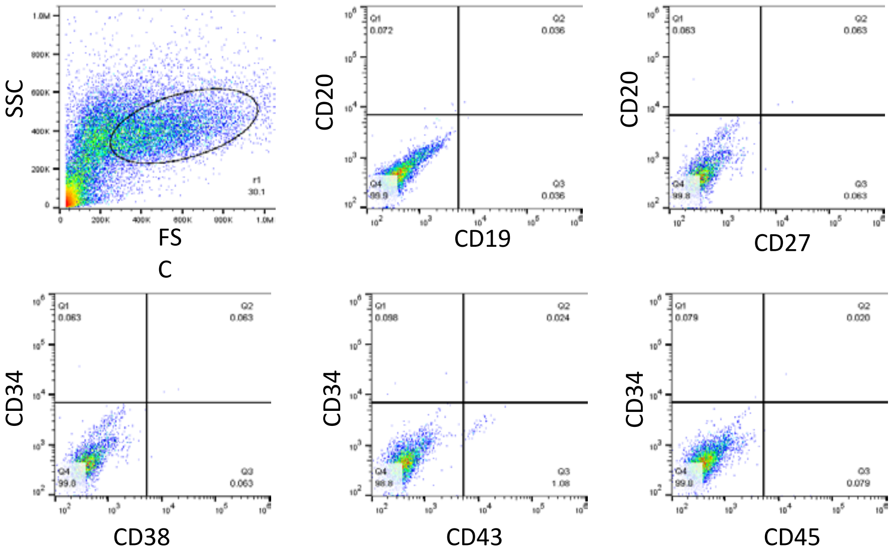
SSC
FS
C
CD20
CD19
CD20
CD27
CD34
CD38
CD34
CD43
CD34
CD45

(b)

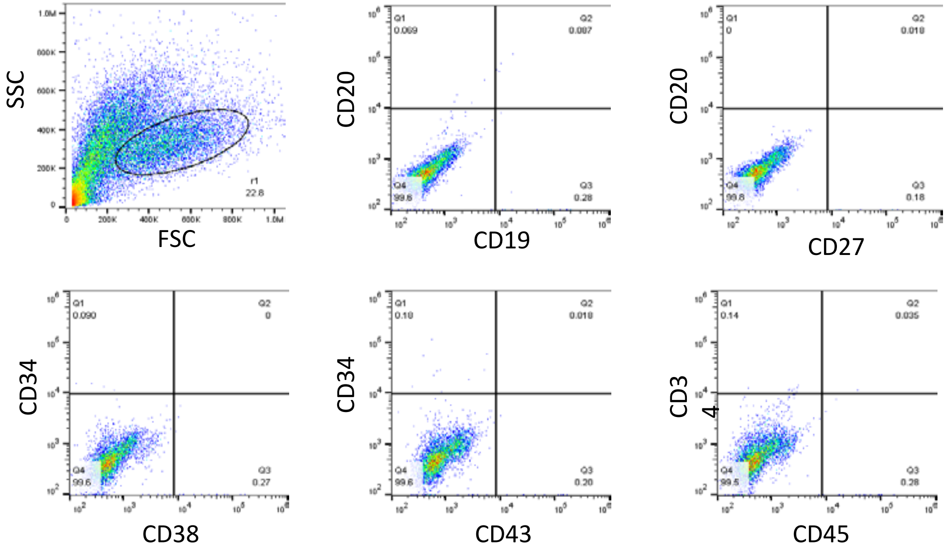
SSC
FSC
CD20
CD19
CD20
CD27
CD34
CD38
CD34
CD43
CD3
4
CD45

Figure S4

(a)

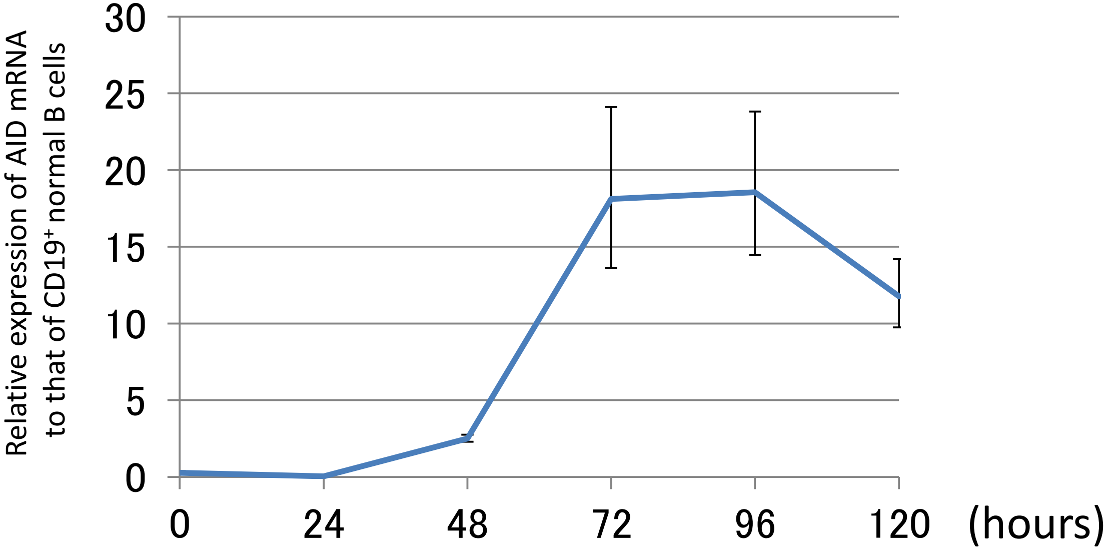
Relative expression of AID mRNA to that of CD19+ normal B cells
30
25
20
15
10
5
0
0
24
48
72
96
120
(hours)

(b)

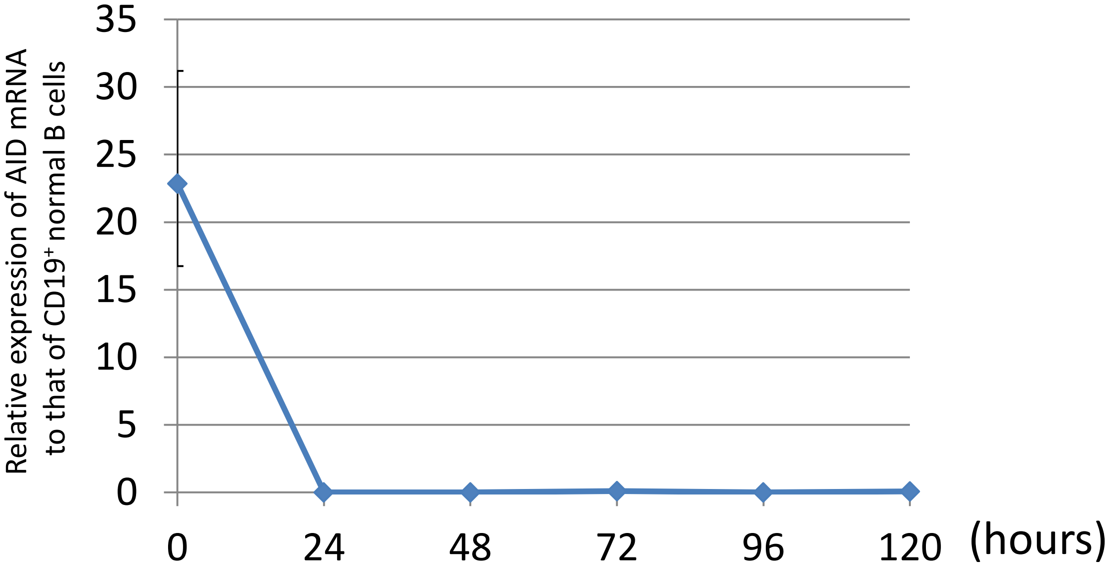
Relative expression of AID mRNA to that of CD19+ normal B cells
35
30
25
20
15
10
5
0
0
24
48
72
96
120
(hours)

Figure S5

(a)

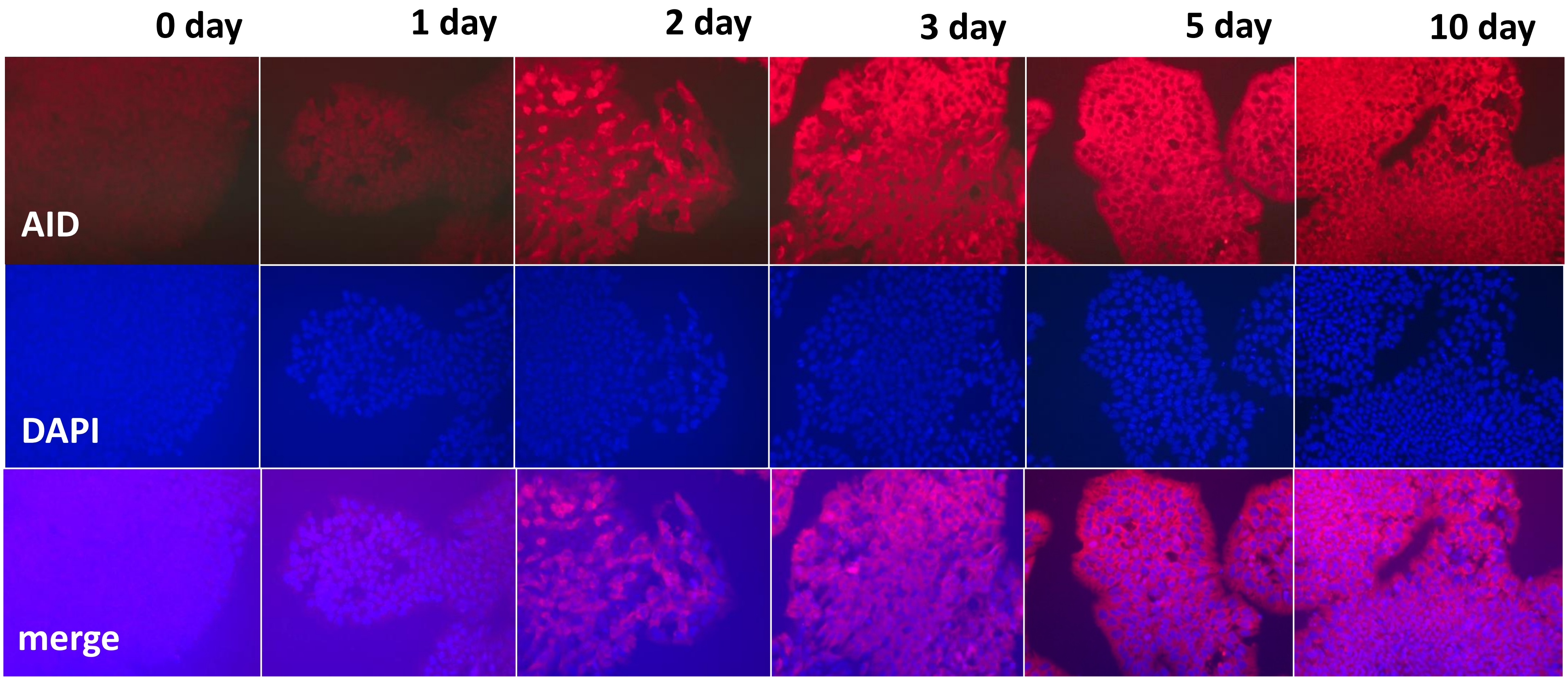

Figure S5

(b)

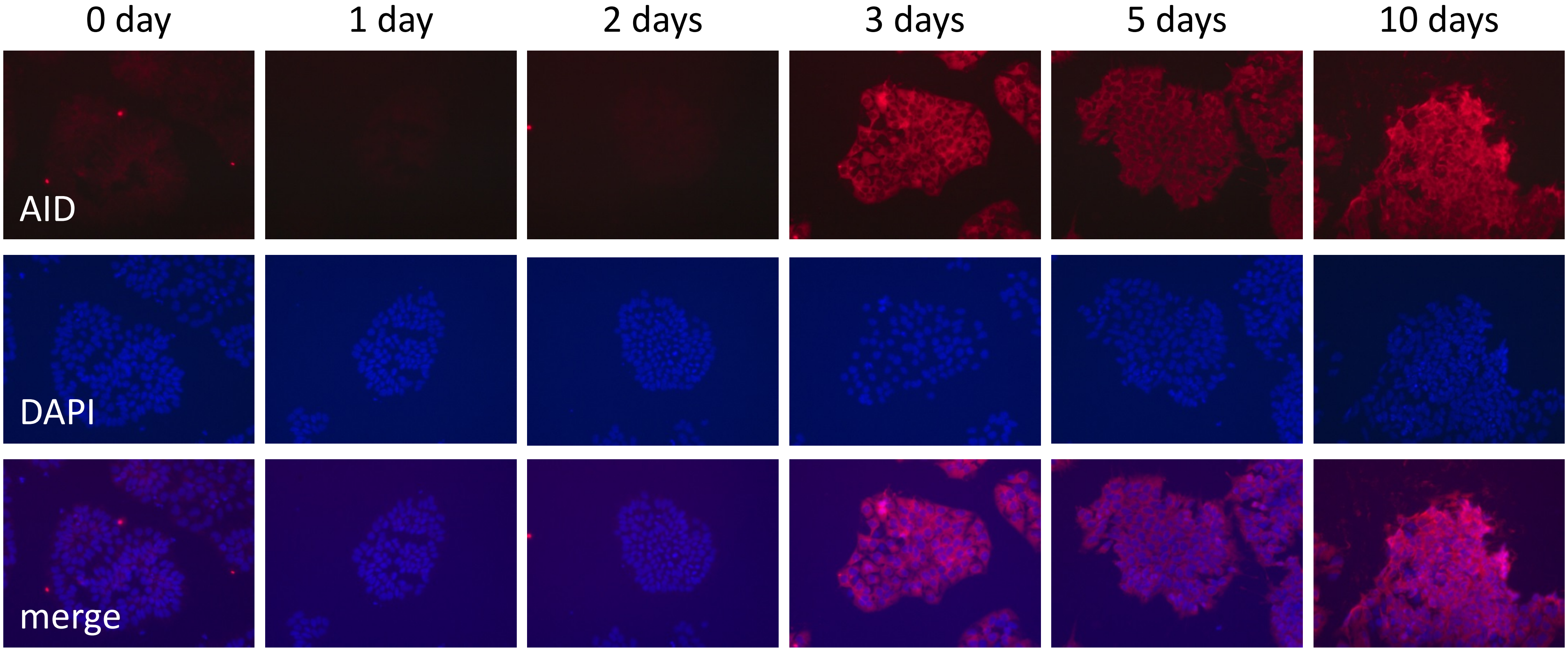

Figure S6

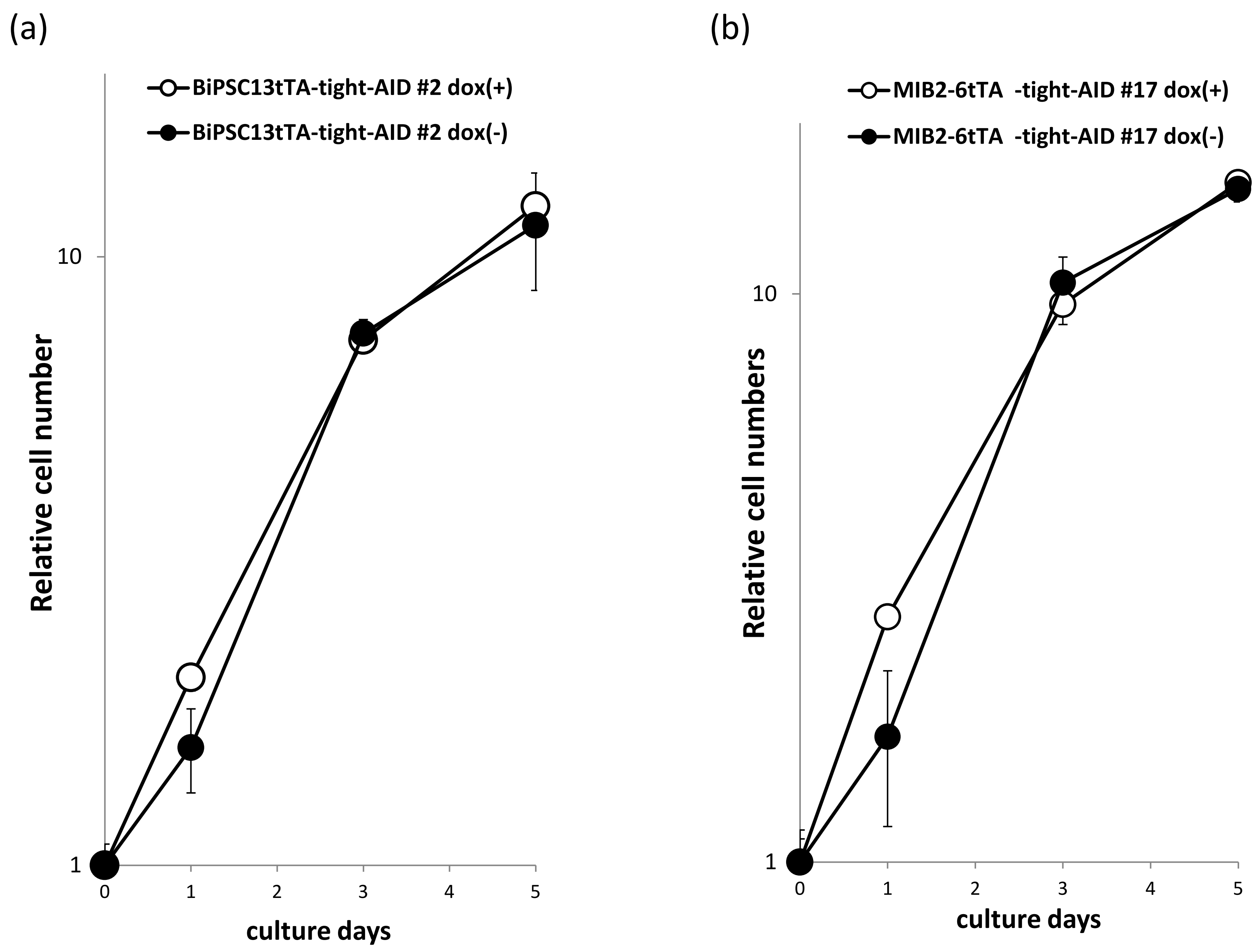
(a)
BiPSC13tTA-tight-AID #2 dox(+)
BiPSC13tTA-tight-AID #2 dox(-)
Relative cell number
10
1
0
1
2
3
4
5
culture days
(b)
MIB2-6tTA -tight-AID #17 dox(+)
MIB2-6tTA -tight-AID #17 dox(-)
Relative cell numbers
10
1
0
1
2
3
4
5
culture days

Figure S7

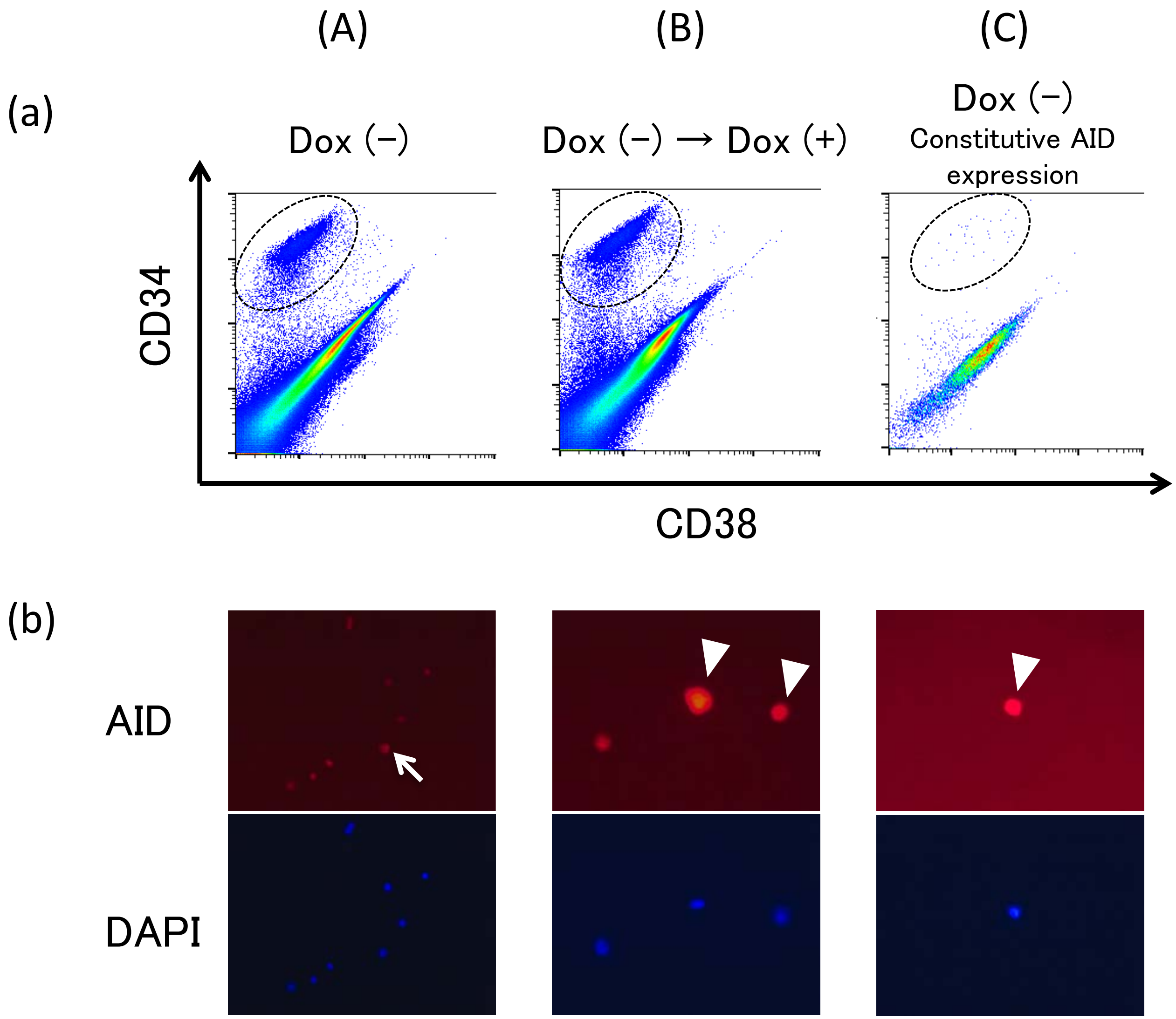
(A)
(B)
(C)
(a)
Dox (−)
Dox (−) → Dox (+)
Dox (−)
Constitutive AID
expression
CD34
CD38
(b)
AID
DAPI
